# Non-Cement-Based Soil Stabilization Material: A Review of Biochar, Nanocellulose, and Recycled Polyethylene Terephthalate (PET) Powder Composite for Sustainable Geotechnics

**DOI:** 10.3390/ma19081598

**Published:** 2026-04-15

**Authors:** Darlington Hyginus Nwaiwu, Dagan Lin, Xiao Wei, Fushen Liu

**Affiliations:** 1 Research Center of Coastal and Urban Geotechnical Engineering, Zhejiang University, Hangzhou 310058, China; 12312186@zju.edu.cn (D.H.N.); weixiaos@zju.edu.cn (X.W.); 2 Zhejiang Key Laboratory of the Development and Utilization of Underground Space, Zhejiang University, Hangzhou 310058, China; 3 Computing Center for Geotechnical Engineering, Zhejiang University, Hangzhou 310058, China; 4 ZCCC Underground Co., Ltd., Hangzhou 310051, China; dagan_lin@163.com

**Keywords:** green soil stabilization, eco geotechnics, waste valorization, carbon sequestration, biobased materials

## Abstract

Soil stabilizers using conventional cement and lime binders incur high environmental costs owing to CO_2_ emissions associated with their excavation, production, and processing. This has motivated research on low-carbon, waste-derived alternatives. The review shows that: biochar increases unconfined compressive strength (UCS) by 15–40% with a 2–5% dosage through pore filling and particle binding; nanocellulose promotes soil cohesion by 25–60% through fibrous network development and tensile bridging; recycled PET powder at 5–10% increases shear strength by 20–35% promoting mechanical interlocking, increasing stiffness, crack resistance and durability. Biochar provides direct carbon sequestration with a carbon transfer capacity of up to 2.5 tons CO_2_-eq/ton. Recycled PET introduces waste valorization, with the potential to divert millions of tons of annual PET waste, while nanocellulose provides indirect carbon savings by avoiding emissions from cement and lime replacement. This review’s objectives are as follows: providing a comprehensive comparison of biochar, nanocellulose, and PET powder as promising non-cement composite stabilizers; identifying optimal dosage ranges and stabilization mechanisms for each material across different soil types; and outlining knowledge gaps and future research directions in sustainable geotechnical practices. The review assessed the individual and synergistic effects of the additives on critical geotechnical properties, including unconfined compressive strength (UCS), California bearing ratio (CBR), resilient resistance, swelling resistance, and the durability of the treated soil. Findings provide actionable guidance for practitioners seeking to reduce construction carbon footprints while maintaining geotechnical performance standards. Research gaps were identified, and future directions for integrating high-performance, low-carbon soil composites into sustainable construction solutions are proposed.

## 1. Introduction

The ever-increasing global demand for infrastructure, transportation networks, and urban development has created an immense need for soil stabilization technologies. With the rapid growth of metropolitan development and increased land use in cities, these soils often require modification if they exhibit insufficient strength, compressibility, hydraulic imbalance, or instability, to ensure reliable and safe construction. As the world population continues to increase, the need for infrastructure development also increases. According to United Nations estimates, between 2018 and 2050, the world’s urban population is projected to grow by 2.5 billion, with approximately 170,000 people added daily [1]. Conventional soil stabilization methods mainly use cement and lime, which are beneficial for maintaining soil strength, stiffness, and durability [2]; however, these binders are associated with high environmental costs. The production of cement alone accounts for significant CO_2_ emissions, with estimates of approximately 0.9 tons of CO_2_ per ton of cement. Research indicates that the cement industry contributes approximately 5–8% of the total CO_2_ emissions worldwide [3]. Thus, the largest share of soil stabilization emissions arises from cement use [4], owing to quarrying, energy requirements, transportation, and raw material processing [5]. Since the construction industry accounts for a significant fraction of global CO_2_ emissions, the use of high-emission stabilizers raises concerns regarding sustainability [4]. With increasing awareness of climate change, high-emission stabilizers are considered unsustainable for broader commercialization [3]. Consequently, several studies have explored the partial replacement of cement with industrial byproducts, bio-based materials, and recycled waste materials. Fly ash, ground granulated blast-furnace slag (GGBS), silica fume, rice husk ash (RHA), and other bio-based materials have been incorporated into soil stabilization systems to enhance durability and engineering performance while reducing carbon emissions. For example, the controlled application of fly ash may improve soil compaction, reduce plasticity, and enhance long-term strength under suitable curing conditions [6,7]. However, a high replacement value may lead to deterioration in cohesion or unconfined compressive strength (UCS), emphasizing the need to maintain optimal mixing ratios to balance environmental benefits and mechanical performance. Studies demonstrate that despite the benefits of cement-based soil additives and other high-energy materials, their inability to achieve net emission reductions on a significant scale is a major constraint [8]. Evidence suggests that biomaterial-based stabilizers have gained attention for addressing waste management issues and sustainable ground improvement. The world produces more biowaste from agricultural, manufacturing, and municipal activities than ever before. Most of this waste is incinerated or disposed of in landfills, generating environmental pollution and greenhouse gas (GHG) emissions. This waste can be recycled into valuable soil stabilization additives [9].

This critical environmental concern has prompted significant research on low-carbon, waste-derived alternatives. Among these, biochar, nanocellulose, and recycled polyethylene terephthalate (PET) powder have emerged as particularly promising sustainable stabilizers, which will be reviewed here. Biochar is a promising bio-based material. Numerous studies have reported the performance of biochar in sustainable soil stabilization. It is produced by the pyrolysis of organic waste under low or no-oxygen conditions. Desirable properties include carbon sequestration, high porosity, variable surface chemistry, and long-term stability. Although biochar has been utilized in agriculture to enhance soil fertility, water retention, and nutrient cycling, recent geotechnical studies have investigated its use for soil stabilization. For instance, incorporating biochar into expansive clays has been shown to reduce compressibility, void ratio, yield strength, and swelling characteristics, thereby making the soil more suitable for road subgrade or foundation applications [10]. Other advantages of biochar include its impact on pore structure, moisture retention, and hydraulic conductivity [10]. The most promising property is the potential for carbon-negative soil stabilization because biochar can sequester carbon for extended periods, depending on the production and activation methods [11].

Cellulose-based materials are also emerging as reinforcement additives for soil stabilization. These include microcrystalline cellulose (MCC), microfibrillated cellulose (MFC), cellulose nanofibers (CNF), and cellulose nanocrystals (CNC), which possess high tensile strength, large aspect ratios, strong interparticle bonding potential, and improved soil structure [12]. They are renewable, readily available, biodegradable, and have low environmental impact. In soil and composite applications, 3D networks of cellulose fibers or nanofibrils form within soil particles, enhancing interparticle contact, closing voids, and increasing resistance to deformation under loading. In addition, large surface areas and hydrophilic functional groups increase moisture content and plasticity, thereby improving ductility and reducing the soil’s shrink–swell characteristics [13]. Collectively, these mechanisms position cellulose-based materials as sustainable, bio-based reinforcement additives for low-carbon and environmentally friendly soil stabilization methods.

Recycled PET powder was selected due to its environmental sustainability, strong mechanical properties, and applicability as a micro-scale soil stabilizer. It helps reduce plastic waste pollution, improves particle packing, load distribution, and durability in fine-grained soils. PET is also hydrophobic, thereby limiting water infiltration and enhancing resistance to shrink–swell behavior [14]. They can also be mass-produced using current recycling processes. Concerns such as microplastic leaching are discussed in Section 4. Using mechanical treatments such as grinding or shredding to form PET powder or small pellets, this waste can be transformed into a valuable additive for soil improvement. Several geotechnical experiments have shown that PET additives enhance the stiffness, tensile strength, and crack resistance of soil, as well as its characteristic lightweight and reinforcement properties [14]. Studies have demonstrated substantial improvements in strength and durability when PET powder was incorporated into clay soils, with marked increases in bearing capacity and reduced deformation under loads [15]. Research also indicates that adding granulated PET waste to sand dunes increased the internal friction angle and shear strength, thereby improving soil stability [16]. Given PET’s chemical stability and resistance to biodegradation under soil conditions, its long-term stability maintains reinforcement effects over an extended service lifespan, thus minimizing maintenance requirements [16]. The PRISMA standard for literature selection and synthesis was adopted to ensure a systematic and transparent review process [17].

The core objectives of this review include (a) providing a comprehensive comparison of biochar, nanocellulose, and PET powder as promising non-cement composite stabilizers; (b) seeking out the optimal dosage ranges and stabilization mechanisms for each material in different soil types; and (c) outlining knowledge gaps and future research directions in sustainable geotechnical practices. To meet these objectives, the review aims to answer the following questions and test the following hypotheses. (i) How do biochar, nanocellulose, and recycled PET powder compare in terms of their physicochemical characteristics, stabilization mechanisms, and effectiveness as non-cement soil stabilizers? Is there a potential for synergy? (ii) What are the optimal dosage ranges and stabilization mechanisms of biochar, nanocellulose, and recycled PET powder for improving the geotechnical properties of different soil types? (iii) What knowledge gaps, research limitations, and future directions exist for the use of biochar, nanocellulose, and recycled PET in sustainable soil stabilization?

Table 1 shows some sustainable supplementary materials that have been explored by other researchers as alternatives to traditional cement and lime for soil stabilization.

## 2. Materials and Methods

The materials selected for this review are biochar, nanocellulose, and recycled PET powder. A comprehensive literature review was conducted to examine their individual performance and potential synergistic interactions in the composite soil matrix. These performances were evaluated across soils of diverse characterizations and types, including clayey, sandy, silty, and fine-grained mixed soils. The physical and chemical properties of the additive materials also vary and are dependent on the laboratory conditions of the reviewed articles. The review follows the Preferred Reporting Items for Systematic Reviews and Meta-Analyses (PRISMA) guidelines to ensure systematic and clear reporting of the literature review [17]. This methodology was chosen because it has broad application in engineering and materials science, and also includes a detailed checklist for methodological soundness and suitability for both quantitative and qualitative syntheses. The results section, which covers the main findings of the review, is shown in Section 3. Section 4 is the discussion, which reviews how these findings relate to geotechnical sustainability expectations. Finally, Section 5 is the conclusion, covering a summary of the review, its limitations, and knowledge gaps. A narrative review approach was used rather than a quantitative meta-analysis, meaning the review did not statistically combine or synthesize datasets from different studies; instead, it explores and interprets existing research and how it relates to the overall review objectives. This is due to the heterogeneity of soil types, additive sources, processing methods, dosage range, specimen preparation, curing conditions, and testing protocols.

### 2.1. Information Sources and Search Strategy

The literature search was conducted using Google Scholar and Google Search. This method ensures that findings cover all publicly available data, including peer-reviewed journals, early-access publications, technical reports, and other interdisciplinary publications on sustainable soil stabilization. Google Scholar and Google Search often index diverse scholarly, professional, and academic sources across geotechnical engineering, materials engineering, environmental engineering, and sustainability. Publications were limited to those published between January 2010 and December 2025 to include early and new developments in low-carbon and waste-derived soil stabilization technologies. Search queries were generated by means of controlled keywords.

The main search strings include key terms such as “biochar soil stabilization,” “activated biochar geotechnical,” “nanocellulose soil reinforcement,” “cellulose nanofibers soil stabilization,” “cellulose nanocrystals soil improvement,” “recycled PET soil stabilization,” “plastic powder geotechnics,” “polymer waste soil reinforcement,” “low-carbon soil stabilizers,” “sustainable geotechnical materials,” and “composite soil reinforcement.” These search terms were combined using “AND” and “OR” to enhance the search results and retrieval accuracy. Relevant publications were selected from known scientific journals and established proceedings at conference meetings. Subsequently, matching journals were assessed to confirm their publication quality, methodology, and relevance to geotechnical engineering applications before being screened for eligibility assessment.

### 2.2. Eligibility Criteria

Only studies meeting a given set of criteria were included in this review, in order to ensure scientific rigor, relevance to geotechnical engineering practice, and consistency in comparative analysis. These include studies discussing the use of biochar, nanocellulose, recycled polyethylene terephthalate (PET) powder or their combinations, either in the context of soil stabilization or geotechnical reinforcement; studies reporting quantitative mechanical, durability, or hydraulic performance such as unconfined compressive strength (UCS), California bearing ratio (CBR), resilient modulus, shrink–swell behavior, permeability, and freeze–thaw resistance; and studies having material characterization data, e.g., scanning electron microscopy (SEM), X-ray diffraction (XRD), surface area analysis and physicochemical methods to facilitate mechanistic interpretation of the reinforcement mechanisms, studies reporting original experiments, field-based or mechanistic data to ensure reliability of the evidence and studies published in peer-reviewed scientific journals or reported in reputable conference proceedings.

In order to minimize uncertainty in the performance comparison of studies, articles lacking standardized testing procedures or well-documented methodologies were excluded. Among these are: studies that were on agricultural soil amendments and unrelated to geotechnical engineering performance evaluation, polymer composites that were not incorporated into soil systems, or about cement-predominant stabilization systems in which biomaterials could be considered only as minor supplementary additives without independent evaluation, papers with insufficient methodological description, quantitative performance-test results, and material characterization, and opinion papers, editorials, and policy documents based on non-technical reports.

Literature reviews and meta-analyses were used for the contextualization, theoretical justification, and identification of pertinent primary sources from the literature. They were not incorporated in the performance synthesis unless they featured consolidated and verifiable primary data that were useful for comparison. The eligibility criteria guarantee high-quality, relevant, and methodologically consistent reviews to support rigorous evaluation of biochar, nanocellulose, and PET-based soil stabilization systems.

### 2.3. Screening and Data Extraction Process

Relevant data were systematically extracted from each eligible study using a standardized data-recording template created to ensure uniformity, comparability, and completeness across the articles. This rigorous, systematic method allowed for reliable integration of experimental results, including evaluation of performance trends and reinforcement mechanisms across multiple studies.

#### 2.3.1. Identification Phase

Google Scholar and Google Search were employed as the primary data sources. The initial search resulted in a total of 1400 results, including 1320 records found on Google Scholar and 80 records found on Google Search. In total, 420 duplicate records were found and removed, leaving 980 unique records for title and abstract screening.

#### 2.3.2. Screening Phase

At this stage, 610 records were excluded due to indirect relevance to soil stabilization, lack of geotechnical performance evaluation, or a primary focus on less relevant applications. The remaining 370 records were subjected to full-text assessment for eligibility.

#### 2.3.3. Eligibility Phase

At this stage, 200 articles were excluded due to inadequate quantitative evidence, an overly strong focus on cement-based stabilization technologies, insufficient experimental procedure description and material characterization, or limited relevance to sustainable geotechnical practices.

#### 2.3.4. Included Studies

**Figure 1:**
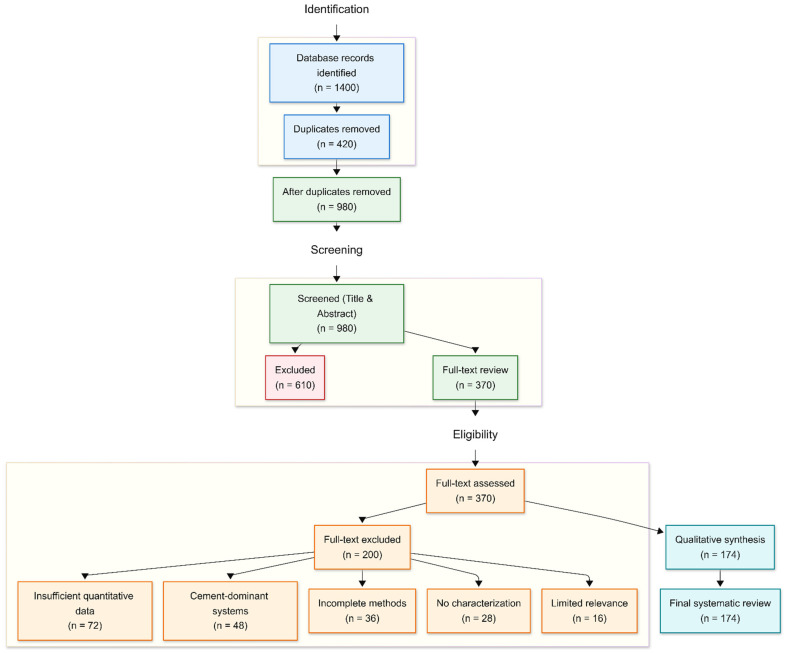
PRISMA flow diagram illustrating the systematic literature selection process adopted in this review using Google Scholar and Google Search.

After rigorous screening, 174 papers met the selection criteria and were then preserved for qualitative synthesis and comparison. These studies were used as data to evaluate the mechanical performance, durability, environmental effect, and synergistic reinforcement mechanism of biochar, nanocellulose, and recycled PET powder in sustainable soil stabilization. The PRISMA method was used to promote transparency, methodological rigor, and reproducibility of the literature selection process. The PRISMA flow diagram in Figure 1 below illustrates the literature identification, screening, eligibility assessment, and inclusion procedures.

## 3. Results

This section presents the results of the synthesized findings from 174 eligible studies: Section 3.1, Biochar: Production and Activation; Section 3.2, Nanocellulose: Production from Lignocellulosic Biomass; Section 3.3, Recycled PET Plastic Powder: Preparation and Production; Section 3.4, Comparative Analysis on the Morphological Properties of Biochar, Nanocellulose and PET; Section 3.5, Mechanical Properties of Biochar, Nanocellulose and PET; Section 3.6, Potential for Carbon Sequestration in Soil. Section 3.1, Section 3.2 and Section 3.3 systematically review the implications of material origin, preparation methods, physicochemical structure, and microstructural properties on the stabilization mechanisms in soil systems. It shows review findings on biochar pyrolysis and activation processes, nanocellulose extraction and fibrillation techniques, and recycled PET powder production via mechanical milling and cryogenic grinding. Differences in feedstock type, thermal processing, physical and chemical activation, crystallinity, and particle size distribution are reviewed, as these factors directly affect porosity, surface chemistry, morphology, and interfacial bonding properties in soil matrices. Section 3.4 provides a comparative morphological analysis based on SEM and XRD analysis results. Surface topography, pore formation, crystalline arrangement, and particle shape are critically analyzed to determine the structural rationale for soil-additive interaction mechanisms. This section compares activation and processing routes to measurable microstructural properties that control particle interlocking, pore filling, fiber bridging, and adsorption properties.

Section 3.5 synthesize the geotechnical performance results, including enhancements in unconfined compressive strength (UCS), shear strength, stiffness, swelling resistance, bearing capacity, durability against wetting/drying and freeze/thaw cycles, and deformation resistance. The performance results are analyzed in terms of dosage range, soil type, curing conditions, and dispersion efficiency. The variability of the results is also presented to show the effect of structure-property relationships on the stabilization process. Section 3.6 assesses the environmental and carbon footprint effects of the three additives. This includes the carbon sequestration potential of biochar, the waste valorization potential of recycled PET, the indirect carbon reduction potential of nanocellulose, and the combined sustainability benefits of the three additives as composite materials.

### 3.1. Biochar: Production and Activation

The production of biochar is performed through pyrolysis, which involves heating agricultural residues, forestry waste, bamboo biomass, and other organic lignocellulosic feedstocks at temperatures of 300–700 °C with little or no oxygen [31]. During this process, the complex structure of the biomass decomposes into three states: biochar (solid), bio-oil (liquid), and syngas (gaseous).

**Figure 2:**
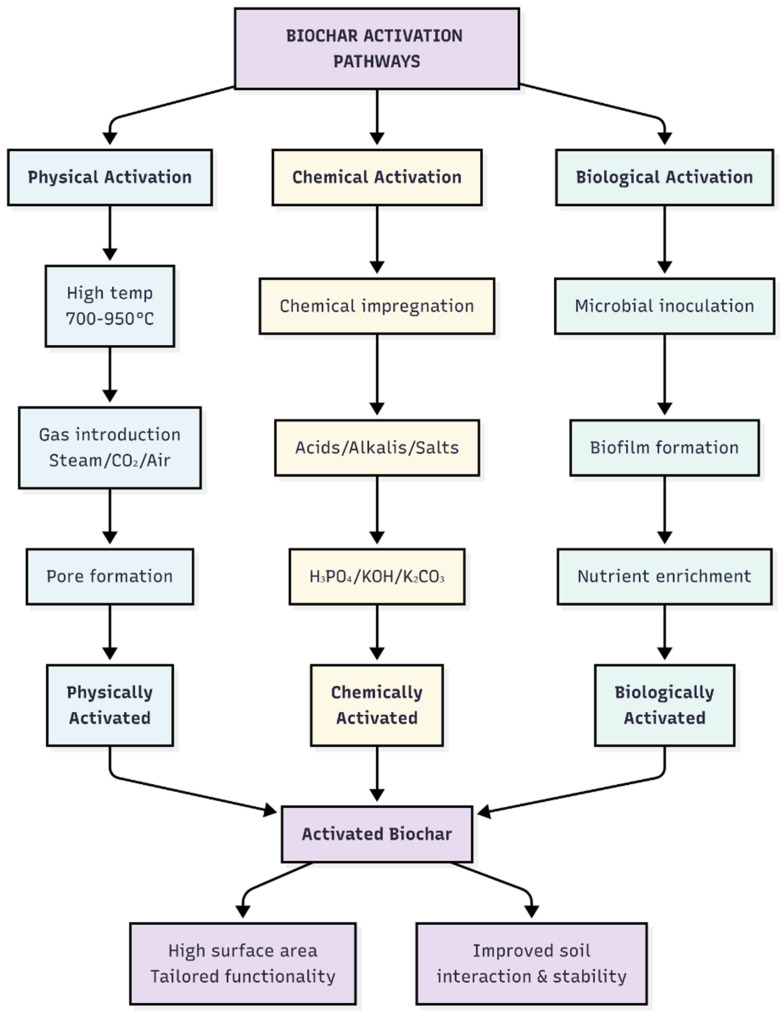
Biochar activation techniques recreated from the source [31].

Pyrolysis plays a crucial role in soil stabilization because it determines the structure and properties of the produced biochar. Biochar produced by low-temperature pyrolysis (300–450 °C) has higher volatile matter and functional groups and can increase the cation exchange capacity and soil–biochar interparticle bonding [32], whereas high-temperature pyrolysis (500–700 °C) produces biochar with increased aromaticity, surface area, thermal stability, and improved degradation resistance, which is adequate for long-term geotechnical purposes [33,34,35]. Pyrolyzed biochar has a porous structure that enhances soil performance by increasing moisture retention, decreasing bulk density, and improving shear strength in expansive soils [35]. Its structure enhances mechanical interlocking with soil particles, and its carbon-rich matrix exhibits excellent stability over a long period of time. After pyrolysis, biochar can be further modified to improve its properties; this is known as activation. Activation greatly increases the surface area, porosity, and functional characteristics of biochar, making it more useful for soil stabilization. Figure 2 shows the physical, chemical, and biological activation methods [31].

Biochar activation is a post-processing treatment that optimizes the physicochemical properties of raw biochar, thereby improving its surface area, porosity, and functional group composition. These enhancements are invaluable in enhancing the effectiveness of biochar as a soil-stabilizing agent, which ultimately determines its interaction with soil minerals, contribution to soil structure modification, and influence on mechanical properties. Physical activation is the most widely adopted and practical method for increasing biochar surface area and porosity [36]. While pyrolysis governs the primary structure of biochar, activation extends its properties to provide additional adaptation features for geotechnical applications, such as swelling control, strength enhancement, and permeability modification, for pavement and foundation systems. Activation is primarily divided into physical, chemical, and biological mechanisms. Physical activation is achieved by exposing biochar to steam or CO_2_ at high temperatures to induce controlled oxidation, thereby promoting pore network growth and increasing the internal surface area [37]. This type of heating yields structurally stable biochar with increased adsorption values and enhanced particle–soil interlocking. The effects of steam or CO_2_ activation on the surface area and pore volume have been reported to be significantly greater than those of raw biochar [38].

Chemical activation employs acids, alkalis, and metal salts to form or modify functional groups, remove mineral impurities, and promote high micro and mesoporosity at relatively low temperatures. Phosphoric acid (H_3_PO_4_), sulfuric acid (H_2_SO_4_), hydrochloric acid (HCl), alkalis—such as potassium hydroxide (KOH) and sodium hydroxide (NaOH)—and metal salts—such as potassium carbonate (K_2_CO_3_), sodium carbonate (Na_2_CO_3_), zinc chloride [ZnCl_2_], and ferric chloride [FeCl_3_]—react with the carbon matrix to etch the structure, thereby increasing the pore volume and surface area, as well as the presence of reactive functional groups on the biochar surface [39]. Acid-activated biochar frequently exhibits a high cation exchange capacity, greater surface functionalization (e.g., oxygen-containing groups), and enhanced affinity for clay or mineral particles in soils. These properties lead to stronger physicochemical bonding and increased soil–biochar interactions in the soil. Alkali activation by KOH treatment has been demonstrated to yield biochar with very high Brunauer–Emmett–Teller (BET) surface areas—in some cases up to >1000 m^2^/g—and well-developed microporosity, enabling substantial improvements in adsorption capacity, porosity, and reactive surface area [39,40]. Salt activation can further adjust the pore size distribution or introduce additional surface chemical modifications, thereby producing biochar with greater versatility for soil amendment [39]. Emerging biological activation strategies employ microorganisms, enzymes, and composting processes to deposit organic coatings, biofilms, and nutrient complexes on biochar surfaces. For example, these bio-engineered or compost-mediated biochar treatments can be performed by microbial activity during composting or inoculation, which modifies the surface chemistry and enriches humic or organic matter–derived functional groups, thus promoting the biodegradability of the biochar with soils and long-term biogeochemical interactions [41]. Biologically activated biochar enhances soil–biochar bonding and aggregate stability, thereby supporting soil stabilization, particularly in environmentally sensitive and marginal soils. Therefore, biochar activation significantly improves its efficacy and flexibility as a sustainable soil additive for carbon sequestration in geotechnical applications. By employing different activation methods, biochar can be tailored to meet diverse soil stabilization requirements, ranging from improving mechanical properties in soils to enhancing long-term durability and soil structural properties. As interest in bio-based soil stabilizers increases, activated biochar offers a new possibility for designing engineered, highly functional, low-carbon, and high-performance solutions for geotechnical and environmental applications. Section 2 discusses in detail the various activation methods used in biochar processing. Figure 2 shows the different biochar activation techniques.

#### 3.1.1. Physical Activation

Physical activation is a commonly used method for improving the structural and surface characteristics of biochar without the addition of chemical reagents, such as KOH and HCL, among others. This method is based on high-temperature gasification reactions between the carbon matrix and oxidizing gases, such as steam, carbon dioxide (CO_2_), or air, via the air-activation method. The formation of an extensive internal pore network, an increase in surface area, and changes in aromaticity strongly influence the effectiveness of the soil-stabilization properties of biochar. During physical activation, raw biochar is heated to 700–950 °C in the presence of an activating gas to create pores within the carbon structure. The gas reacts with the carbon surface by etching disordered carbon phases, thereby promoting pore formation and expansion. The controlled oxidation process increases the microporosity and mesoporosity, thereby preserving the mechanical structure of biochar [36]. This porous framework facilitates interactions with soil particles, regulates moisture, and promotes particle interlocking, which are critical for stabilizing subgrade soils, expansive clays, and weak foundation soils.

(a) Steam Activation

Steam activation is widely used for biochar activation because it effectively promotes pore formation, thereby improving soil water retention capacity and promoting long-term stability. Steam reacts with carbon, releasing hydrogen and carbon monoxide, which, during this process, create pore voids. Steam-activated biochar typically has a high surface area and water retention capacity, which are very useful for stabilizing soil in arid environments and improving soil mechanical properties [36,42]. The chemical reactions involved in steam activation are shown below.

C + H_2_O → C(O) + H_2_ (1a)

C(O) → CO + C(1b)

C + 2H_2_O → CO_2_ + 2H_2_ (1c)

C + CO_2_ → 2CO (1d)

C + 2H_2_ → CH_4_ (1e)

CH_4_ + H_2_O → CO + 3H_2_ (1f)

Steam activation is achieved through high-temperature gas–solid reactions between carbon and water vapor, which gradually gasifies carbon, creating pore voids. The carbon initially reacts with steam to form oxygenated surface complexes and hydrogen, as seen in Equation (1a), then decomposes to release carbon monoxide and regenerate reactive carbon sites (1b). Subsequently, carbon monoxide undergoes a water–gas shift reaction with steam to produce carbon dioxide and hydrogen (1c). Simultaneously, steam directly gasifies carbon, releasing carbon dioxide and hydrogen gases (1d). The carbon dioxide produced reacts with residual carbon through the Boudouard reaction, releasing more carbon monoxide, which creates etching of the carbon surface (1e). Methanation and steam reforming reactions can also occur under hydrogen-rich conditions (1f). The gases released during this process progressively enlarge the pores, thereby increasing the surface area and reactivity of the steam-activated biochar [36,42].

(b) CO_2_ activation

CO_2_ activation is a standard method for increasing the porosity and surface area of biochar. Biochar is heated at high temperatures of 800–950 °C to react with CO_2_ gas through the Boudouard reaction [36,43]. The chemical reaction involved in this process is the same as Equation (1e). This reaction partially gasifies the carbon, removing reactive carbon atoms and forming a more porous structure. Progressive carbon removal creates micropores and mesopores, increasing the surface area and enhancing the adsorptive performance of biochar. Additionally, CO_2_ activation indirectly creates reactive sites, such as edged carbons and oxygen-containing groups, which enhance surface functionality through chemical reactivity and the material’s ability to react with adsorbates or catalysts [36,43].

(c) Air Activation Methods

This method uses air as an oxidizing agent, relying on the strong oxidizing properties of oxygen. Because pure oxygen is expensive, air is typically used in this process. The mechanism involves several reactions, as shown below in (2a) and (2b) [36].

2C + O_2_(g) → 2CO(g) (2a)

C + O_2_(g) → CO_2_ (2b)

Air oxidation introduces oxygen-containing functional groups, such as –COOH, –OH, and C=O, into biochar, thereby enhancing its hydrophilicity and chemical reactivity; however, pore blocking during this process can slightly reduce the surface area [36,44]. When applied prior to pyrolysis, usually at low temperatures of 170–310 °C, it promotes the incorporation of oxygen, develops pore structures, preserves the morphology of the feedstock, and enhances biochar performance. Air pre-oxidation greatly enhances pore formation, particularly after an activation process, which could be chemical or physical CO_2_ activation. The chemically adsorbed oxygen can increase the microporosity and BET surface area of the material by helping create micropores. The meso-porosity can also develop up to 44% of the total pore volume through the decomposition of oxygen functional groups during reactivation, which leads to the enlargement of existing pores [36,44]. Table 2 below shows physically activated biochar derived from various feedstocks.

#### 3.1.2. Chemical Activation

Chemical activation methods involve treatment with acids, alkalis, or metal salts to improve pore formation and surface chemistry of the biochar. During chemical activation, the surface area and specific density of the biochar increase significantly compared to those during physical activation [39,53]. The activation of biochar using acids, such as phosphoric acid (H_3_PO_4_), sulfuric acid (H_2_SO_4_), or hydrochloric acid (HCl), introduces more oxygen-bound functional groups, increasing the surface acidity and opening new pathways for pores within the biochar. This increases the binding capacity and cation exchange property of the biochar [53,54].

Alkali activation, often from potassium hydroxide (KOH) or sodium hydroxide (NaOH), selectively etches the carbon matrix, yielding highly microporous structures with high surface area [39], and these alkali-activated biochars are very reactive particles that can pozzolanically interact with the soil minerals, increasing soil strength and structural stability as well [55].

Salt-activated biochar using metal salts, such as potassium carbonate (K_2_CO_3_), sodium carbonate (Na_2_CO_3_), zinc chloride (ZnCl_2_), and ferric chloride (FeCl_3_), improves the bioactive adsorbent property of soils contaminated with heavy metals and organic compounds [55]. During metal salt activation, the metal salt penetrates the carbon matrix, removes mineral impurities, and forms micro and mesopores as well as introducing new hydroxyl, carboxyl, and phosphate groups, thereby enhancing the surface polarity and chemical interactions with soil minerals [39,53].

Chemically activated biochar exhibits a high surface area and strong adhesion to clay and silt particles, thereby improving water-holding capacity and drainage control, as well as potential pozzolanic reactivity when added to lime or cement [53,54]. The resulting biochar improves long-term durability in soil subgrade and foundation reinforcement applications.

(a) Acid Activation

Acid activation is used to improve the surface properties and reactivity of biochar. In this process, biochar is treated with strong acids, such as phosphoric acid (H_3_PO_4_), sulfuric acid (H_2_SO_4_), or hydrochloric acid (HCl), which penetrate the carbon matrix, eliminate mineral impurities, and increase porosity. Acid activation increases the surface acidity of biochar and introduces oxygen-containing functional groups, such as carboxyl, hydroxyl, and phosphate groups, which are essential for promoting chemical interactions between biochar and soil particles [53,54]. Acid-activated biochar typically exhibits a higher specific surface area and greater micro and mesoporosity than raw biochar. These structural changes may enhance water retention, cation exchange, and adhesion between biochar and clay-silt particles, thereby increasing the ability to stabilize expansive, organic, and fine-grained soils [53,54].

Acid-activated biochar properties depend on the type of acid, concentration, treatment duration, activation temperature, and feedstock. For instance, pretreatment with H_3_PO_4_ may markedly enhance the specific surface area and micropore distribution of biochar via acid-catalyzed reactions and phosphate-mediated crosslinking. In addition, acid reactions can enhance the hydrophilicity and porosity of biochar, thereby improving their absorption characteristics [53,54]. Owing to these effects, acid-activated biochar can be adapted for tailored soil stabilization, such as increased water retention, improved interaction with fine soil particles, and long-term durability under cyclic wetting/drying or freeze–thaw cycles; the latter is particularly effective for stabilizing expansive or moisture-sensitive soils. The following Equations (3a)–(5e) explain the acid activation processes.

2P_2_O_5_ + 5C → P_4_ + 5CO_2_ (3a)

2H_2_P_2_O_7_^2−^ → P_4_ + 6O_2_ + 2H_2_O (3b)

During phosphoric acid activation, H_3_PO_4_ decomposes into polyphosphates, which enhance pore growth and add phosphorus-oxygen functional groups, including C–PO_3_, on carbon surfaces. Equation (3a) consists of phosphorus pentoxide reacting with carbon to generate elemental phosphorus and CO_2_, which promotes etching and an increase in porosity. Complementing this, Equation (3b) shows pyrophosphate decomposition into phosphorus, oxygen, and water, thus leading to improvements in structural and surface area properties of the biochar [36].

C_n_H_2n_O_n_ + H_2_SO_4_ → nC + H_2_SO_4_·nH_2_O (4)

Sulfuric acid activation primarily dehydrates organic precursors, such as carbohydrates present in the biomass, to form carbon while removing minerals and enhancing porosity. Equation (4) demonstrates how H_2_SO_4_ extracts water from the organic material, decomposing it to elemental carbon and a hydrated acid complex, which carbonizes the material and also removes ash, forming a mix of large and medium pores, thus improving the CO_2_ adsorption property of the activated biochar [36,54].

HNO_3_ → NO_2_ + O_2_ + H_2_O (5a)

2NO_2_ + C → C(O) + 2NO (5b)

C(O) + H_2_O → COOH (5c)

C + HNO_3_ → C–OH + 2NO_2_ (5d)

C + O_2_ → CO (5e)

Nitric acid activation is performed at moderate temperatures of 25–100 °C, oxidizing the carbon surface and introducing oxygen-containing functional groups, such as carboxyl (–COOH), hydroxyl (–OH), and carbonyl (C=O) groups. These groups enhance the surface acidity and increase the adsorption capacity of the biochar. The activation process starts with the decomposition of nitric acid into reactive elements as seen in Equation (5a), which forms surface oxides (5b). The hydrolysis of these oxides produces carboxyl groups (5c). In contrast, other reactions produce hydroxyl groups (5d), which promote etching of the carbon surface (5e). These reactions produce voids and active sites, thereby enhancing the interaction between biochar and adsorbates [36].

(b) Alkali Activation

Alkali activation considerably increases the porosity, surface area, and reactivity of biochar. In this activation method, biochar is reacted with powerful alkalis, such as potassium hydroxide (KOH) and sodium hydroxide (NaOH), which permeate the carbon matrix and rapidly etch it. The etching results in a densely microporous network with a large surface area [53,54]. The reaction of alkalis with biochar enhances its reactivity and increases the surface area and functional groups, thereby facilitating effective interactions with soil particles. For example, KOH-activated biochar can achieve surface areas of 100–1000 m^2^/g, which is significantly higher than that of untreated biochar, with markedly improved porosity and surface area, as well as substantially greater reactivity. This biochar may be characterized by enhanced surface polarity and oxygen-containing functional groups, thereby enhancing its potential for chemical interactions with soil minerals [55]. These characteristics make alkali-activated biochar a potential candidate for geotechnical and sustainable applications [56]. High microporosity can facilitate water retention and drainage control, which are essential for managing expansive and fine-grained soils. Enhanced surface interactivity and functional groups may enable stronger physicochemical interactions with soil minerals, potentially increasing soil strength, stiffness, and long-term durability when they are appropriately mixed.

The performance of alkali activation relies heavily on alkali type (KOH or NaOH), impregnation ratio, temperature, and reaction duration. For instance, a recent evaluation of rice husk biochar indicated that pre-activation with KOH in a CO_2_ atmosphere led to 3.2- and 30-fold increases in the surface area and pore volume, respectively, in comparison to untreated biochar [57]. Under appropriate activation conditions, such as optimal alkali ratio and temperature, a well-defined microporous structure, high carbon content, and high surface functionality are achieved [58]. Alkali activation is highly efficient at generating highly reactive biochar with excellent porosity and surface chemistry, making it applicable to advanced geotechnical and environmental applications.

The chemical Equations (6a)–(6d) describe the KOH redox reactions and etching processes that generate pores and increase the surface area of the activated carbon structure [36,53].

2KOH + CO_2_ → K_2_CO_3_ + H_2_O (6a)

2C + 2KOH → 2CO + 2K + H_2_ (6b)

K_2_CO_3_ + C → K_2_O + 2CO (6c)

K_2_O + C → 2K + CO (6d)

In these reactions, CO_2_ and H_2_O are produced at high temperatures to react with KOH, forming K_2_CO_3_, as shown in Equation (6a). Simultaneously, the direct reaction between KOH and carbon produces hydrogen (H_2_) and carbon monoxide (CO) gases, as shown in (6b). The release of these gases from biochar creates extensive porous structures. As temperature increases, the newly formed K_2_CO_3_ reacts with carbon to produce additional gaseous products, including metallic potassium and CO, as shown in Equations (6c) and (6d) [36,53]. Activation with NaOH yields a stronger and more sustainable oxidation process. The reaction process is shown below (7a)–(7c) [36].

2C + 6NaOH → 2Na + 2Na_2_CO_3_ + 3H_2_ (7a)

2C + Na_2_CO_3_ → 2Na + 3CO (7b)

2Na + CO_2_ → Na_2_O + CO (7c)

Sodium hydroxide (NaOH) activation generates reactive intermediates, including CO, CO_2_, and H_2_, facilitating the infiltration of Na and Na_2_CO_3_ into the carbon matrix. This mechanism expands the carbon structure, thereby increasing the specific surface area and pore size. However, a very high NaOH dosage can accelerate the gasification reaction and decrease the effective surface area of the biochar. At high concentrations, reactions such as C–NaOH, C–Na_2_CO_3_, C–Na_2_O, C–Na, C–CO_2_, and C–CO can occur, breaking C–C and C–O–C bonds and lowering the carbon yields [36].

(c) Metal Salt Activation

Metal salt activation is a chemical process that employs metal salts like potassium carbonate (K_2_CO_3_), sodium carbonate (Na_2_CO_3_), zinc chloride [ZnCl_2_], and ferric chloride [FeCl_3_] to alter the porosity, surface chemistry, and mineral content of biochar. In this method, a metal salt solution is added to biochar, which is then thermally treated to promote dehydration, carbonization, and pore formation. The metal ions facilitate the formation of a highly porous structure and may possess catalytic or reactive sites on the biochar surface [59].

Metal salt-activated biochar has markedly greater surface area, micropore and mesopore volumes, and chemical activity than raw biochar. For example, active biochar containing ZnCl_2_ exhibited a specific surface area (BET) of 375.9 m^2^/g and total pore volume of 0.17 cm^3^/g, which is almost five times higher than that of raw biochar (78.6 m^2^/g, 0.03 cm^3^/g) after activation. Furthermore, metal salt activation can provide additional adsorption properties and enhanced interactions, as well as soil mineral sorption, impurity removal, and geochemical reactions within biochar [59]. This has been demonstrated for heavy metal removal, including Cr(VI) [59]. Metal salt concentration and activation temperature strongly determine the final biochar performance. For instance, the use of ZnCl_2_ as an activator typically yields high microporosity and a good pore structure because of its dehydrating and volatilizing behavior during pyrolysis [60]. Alternatively, activation with salts such as ZnCl_2_ and FeCl_3_ has been identified as one of the most promising means of increasing biochar’s heavy-metal adsorption capacity, compared to other activation pathways [61]. The following chemical Equations (8a)–(11) show the activation processes of different metal salts.

K_2_CO_3_ → K_2_O + CO_2_ (8a)

K_2_O + C → 2K + CO (8b)

In Equation (8a), K_2_CO_3_, a common activating agent, decomposes at 700–900 °C to produce CO_2_ and K_2_O. CO_2_ serves as a gasifying agent, generating pores in the carbon matrix [36].

Na_2_CO_3_ → Na_2_O + CO_2_ (9a)

Na_2_O + C → 2Na + CO (9b)

Na_2_CO_3_ follows a similar activation process. Upon heating, it decomposes to Na_2_O and CO_2_, as shown in Equation (9a). CO_2_ also serves as a gasifying agent, generating pores in the carbon matrix. Na_2_O then reacts with carbon as shown in (9b), forming elemental sodium that intercalates into the structure of the material. Na_2_CO_3_-activated carbons have a high surface area and improved adsorption properties, making them suitable for sustainable applications [36].

ZnCl_2_ + C → Zn + Cl_2_ + CO (10)

ZnCl_2_ is widely recognized for its strong dehydrating effect. During pyrolysis, it interacts with the biochar, ultimately promoting pore development. Activation with ZnCl_2_ tends to produce more mesopores and macropores due to the deep penetration of molten ZnCl_2_ into the carbon matrix [36].

2FeCl_3_ + 3C → 2Fe + 3Cl_2_ + 3CO (11)

FeCl_3_ is another effective activation agent that primarily promotes the formation of micropores. It acts as a catalyst, removing volatile compounds and facilitating the development of highly porous structures. The resulting carbon materials possess high micropore volumes, making them attractive for gas storage and separation [36]. Table 3 summarizes the properties and CO_2_ adsorption capacities of various chemically activated biochars reported in recent studies.

### 3.2. Nanocellulose: Production from Lignocellulosic Biomass

Nanocellulose is a cellulose-derived nanomaterial characterized by its nanoscale size, high aspect ratio, large specific surface area, and excellent mechanical and chemical properties. It can be derived from lignocellulosic biomass like wood, agricultural residues, wheat straw, rice husks, and other plant-based waste. Bacteria can also be used to produce bacterial nanocellulose (BNC) [74,75]. Lignocellulosic biomass primarily consists of cellulose, hemicellulose, and lignin. Nanocellulose can be extracted using mechanical, chemical, or enzymatic processes to remove non-cellulosic materials and isolate cellulose nanostructures. Nanocellulose is classified into three major categories: cellulose nanocrystals (CNCs), cellulose nanofibrils (CNFs), and bacterial nanocellulose (BNC). CNCs are rigid, rod-shaped crystalline particles; CNFs are long, flexible, and entwined fibrils; and BNCs, from microbial fermentation, can form highly pure and uniform nanofiber networks [74,76].

The mechanical properties of nanocellulose are among the highest of all natural polymers. A good example is the stiffness of CNCs; the Young’s modulus of CNCs has been reported to be between 100 and 170 GPa, and the tensile strength to be approximately 7.5–7.7 GPa [75,77]. These extraordinary mechanical properties are due to their excellent crystallinity and strong hydrogen bonding network [75]. Due to the hydroxyl-rich nature of nanocellulose, strong hydrogen bonding, chemical modifications, and interfacial interactions with polymers, nanocellulose shows significant performance enhancement in composite materials [75,76], through effective stress transfer, increased stiffness, load-bearing capacity, and durability [75,77]. Nanocellulose-based composites offer numerous advantages for geotechnical and soil-stabilization applications. The high aspect ratio and crystallinity improve mechanical strength and stiffness when combined with fillers such as biochar [13]. Additionally, the network-forming properties of CNFs or BNCs can ameliorate microstructural aberrations, reduce filler accumulation, and promote uniformity for homogenization and dispersion, thereby stabilizing soils or composites by strengthening their structural properties [78]. At the same time, the hydrophilic nature and larger surface area of nanocellulose can enhance water stability, reducing shrinkage or cracking in cyclic wetting-drying or freeze–thaw conditions [13]. The reactive hydroxyl groups can form chemical linkages with cementitious or pozzolanic matrices, thereby enhancing durability and bonding properties in soil–biochar–polymer composites [79].

The method of nanocellulose extraction, fibrillation or crystallinity, surface treatment, and mix ratio significantly influence the properties of nanocellulose-reinforced composites. For instance, CNCs provide high stiffness and tensile reinforcement, CNFs provide networked structural support and toughness, and BNCs provide uniform three-dimensional fibrillar structures [74,76]. By carefully considering and tuning these parameters, nanocellulose can be mixed with recycled polymers and biochar to produce sustainable, high-performance composite materials with improved mechanical, thermal, and water absorption properties, which are promising for low-carbon building materials and soil stabilization projects.

#### 3.2.1. Lignocellulosic Biomass

Lignocellulosic biomass consists of a wide range of natural organic materials derived from plants. As one of the most abundant renewable carbon reservoirs on Earth, it is a highly promising feedstock for producing biofuels, bioethanol, and other biochemicals [80,81]. Due to its renewable nature, this material serves as a source of renewable natural fibers that can replace petroleum-based polymers [81]. Additionally, biomass residues such as agricultural and forestry wastes contain lignocellulose, eliminating competition with human or animal food resources [82]. The cell walls of lignocellulosic biomass are mainly composed of three components: cellulose, hemicellulose, and lignin [83,84]. The structural composition of the biomass is dependent on the species and the origin of the biomass [80,85]. Lignin generally contributes 10–25% of the dry weight of lignocellulosic materials [84]. Within plant cell walls, lignin acts as a natural binder, joining the cellulose and hemicellulose matrices. This binding process preserves the mechanical integrity, rigidity, resistance to degradation, water resistance, and impermeability of the cell wall [86,87]. Chemically, lignin is a cross-linked amorphous copolymer formed from phenyl-propane monomers known as monolignols. The chemical structure of lignin varies among plant biomasses due to differences in the degree of methoxy substitution on the aromatic rings [88].

**Figure 3:**
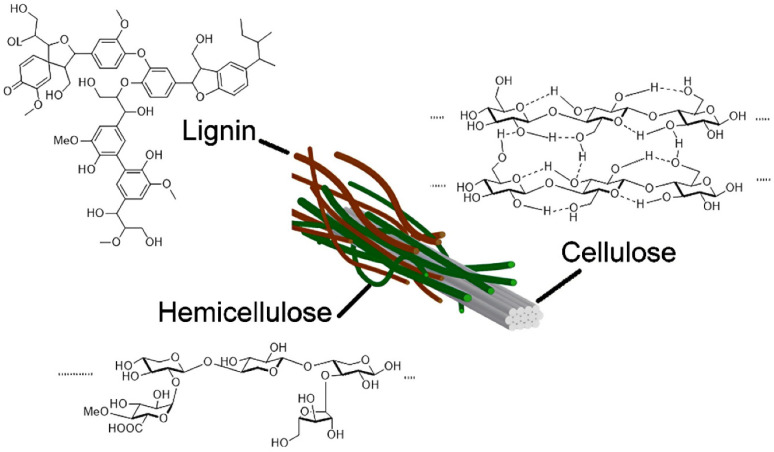
Cellulose, hemicellulose, and lignin inside a ligno-biomass [92].

Lignin, as a precursor, has recently attracted research interest for the separation of lignocellulosic biomass and its depolymerization to produce biofuels and other products [89,90]. For instance, lignin-derived carbon materials are increasingly being used in catalysis, energy storage, and environmental remediation [90,91], demonstrating their versatility and promising prospects. A schematic representation of the molecular structures of cellulose, hemicellulose, and lignin in lignocellulosic biomass is shown in Figure 3.

Hemicellulose accounts for approximately 20–35% of lignocellulosic biomass. It is a heteropolymer composed of short, linear, and branched chains of various sugar monomers, including pentoses, hexoses, and others. Common hemicellulose types include xylans and glucomannans, with xylans primarily occurring in hardwoods and glucomannans being more prevalent in softwoods. Hemicellulose binds to cellulose fibrils via hydrogen bonds and van der Waals interactions, forming cross-links with lignin. The binding of hemicellulose with cellulose and lignin is essential for maintaining the structural integrity and mechanical strength of plant cell walls. Under mild conditions, hemicellulose can be hydrolyzed using acids, alkalis, or enzymes to produce monomers and oligomers that can be converted into bioethanol or high-value chemicals [93,94].

**Figure 4:**
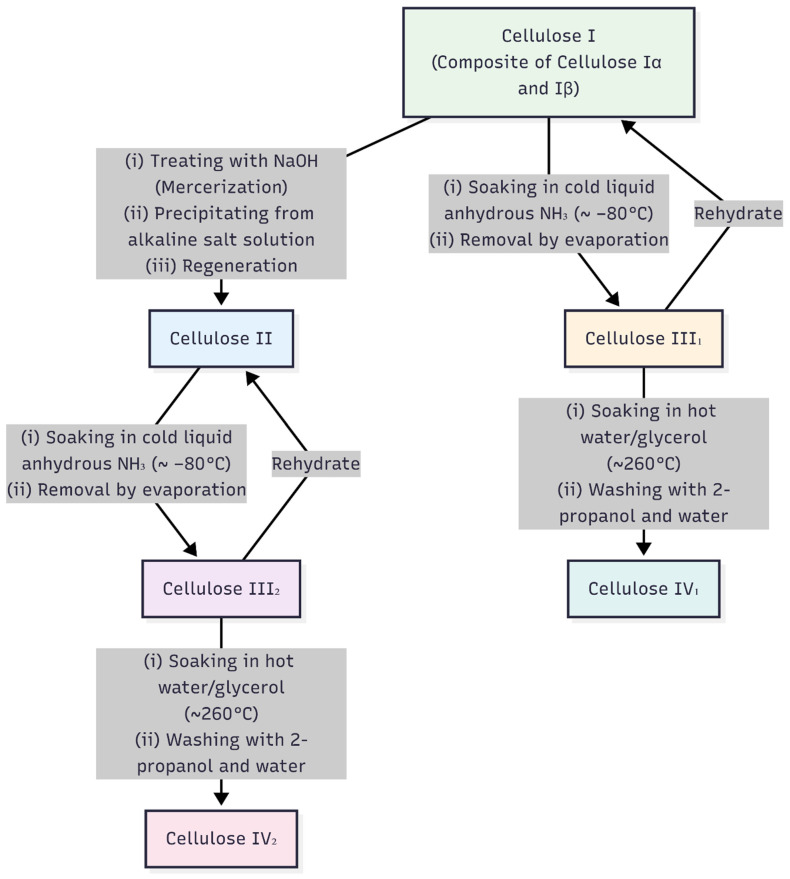
Illustration of the interconversion of cellulose I and cellulose II into other cellulose allomorphs. Reproduced from source [97].

Cellulose is the primary component of lignocellulosic biomass, constituting approximately 35–50% of plant cell walls. It is a linear homopolysaccharide with β-1,4-linked D-glucose units [83,95]. Glucose monomers contain hydroxyl groups that form strong intramolecular and intermolecular hydrogen bonds with the adjacent units. These hydrogen-bond networks form tightly packed crystalline regions within cellulose fibrils, making them mechanically strong, water-insoluble, and resistant to most organic solvents [83,96]. Native cellulose I has a triclinic crystal structure (space group *P*1) and its unit cell contains one cellulose chain with 42 atoms. For comparison, cellulose I is monoclinic (space group *P*21), with two distinct parallel chains per unit cell with 84 atoms. Apart from native cellulose I phases, cellulose exists in several different crystal forms known as allomorphs, namely cellulose II, III_1_, III_2_, IV_1_, and IV_2_, which can be synthesized by the thermochemical treatment of cellulose. The steps involved in the interconversion of different cellulose allomorphs are shown in Figure 4 [97].

Cellulose II is synthesized from cellulose I and has a monoclinic crystal structure (space group *P*21). Its unit cell contains two cellulose chains, which are arranged in an antiparallel manner. Cellulose III_1_ and III_2_ are prepared from cellulose I and II, respectively. Cellulose III_1_ has a monoclinic unit cell, containing one cellulose chain. In cellulose III_1_, chains are arranged in parallel fashion, as in cellulose I, with slightly different conformations. The crystal structure of cellulose III_2_ is not well established yet. Both cellulose III_1_ and III_2_ revert back to their original forms when they are treated with boiling water. Cellulose IV_1_ and IV_2_ are prepared from cellulose III_1_ and III_2_, respectively, by thermal treatment in hot glycerol [97]. Due to its high carbon content and hydroxyl-rich structure, cellulose is a natural resource for carbon-based materials, essential chemicals, composites, textiles, paper, and many other engineering applications [83]. Interconversion pathways between cellulose allomorphs are schematically illustrated in Figure 4. A summary of the typical chemical composition of major cellulosic materials is shown in Table 4.

#### 3.2.2. Methods of Extracting and Producing Cellulose Nanocrystals (CNCs) from Plant Fibers

The simplest and most common method for CNC extraction is acid hydrolysis, in which the amorphous regions of cellulose are selectively hydrolyzed by strong mineral acids, such as sulfuric acid (H_2_SO_4_) or hydrochloric acid (HCl). Sulfuric acid treatment introduces negatively charged sulfate groups on the CNC surface, enhancing its colloidal stability and dispersibility in aqueous media. HCl hydrolysis results in crystallites and low charge on the surface, and therefore is used by applications for which unmodified high-strength nanocrystals are desired [86]. Pretreatment with alkali is frequently performed before acid hydrolysis to remove hemicellulose and facilitate fiber delignification [87]. This can typically be bleached by adding agents such as sodium chlorite or hydrogen peroxide to improve cellulose purification, producing the characteristic white color and crystallinity [88]. Enzymatic pretreatment methods employing cellulases or hemicellulases can selectively degrade amorphous cellulose and reduce energy consumption during hydrolysis, making it environmentally friendly [89].

Chemical treatments are sometimes combined with mechanical methods, including high-pressure homogenization, microfluidization, ultrasonication, and grinding, to minimize fiber size and improve the uniformity of nanocrystals, thereby enabling the production of high-aspect-ratio CNCs [90]. These methods are also beneficial for reducing agglomeration and increasing dispersibility within polymer or biochar matrices [91]. CNCs from plant fibers are usually 100–500 nm long and 3–20 nm wide, depending on the cellulose source and hydrolysis conditions [83,92]. CNCs are considered an effective reinforcement additive for low-carbon composites due to their rod-shaped morphology, high crystallinity of >70–80%, good mechanical properties, tensile strength of 7.5–7.7 GPa, and Young’s modulus up to 150 GPa [93,94]. CNCs can be tailored to specific mechanical, thermal, and water-tolerant properties by optimizing the biomass source, pretreatment conditions, acid concentration, hydrolysis conditions, and surface functionalization, making them suitable for inclusion in biochar-reinforced recycled polymer composites for soil stabilization and environmentally sustainable construction [95].

#### 3.2.3. Methods of Extracting and Producing Cellulose Nanofibers (CNFs) from Plant Fibers

Cellulose nanofibers (CNFs) are nanoscale fibers with high mechanical strength, flexibility, and large surface area. CNFs preserve the crystalline and amorphous sections of cellulose; thus, they possess a combination of stiffness and network properties. The extraction of CNF from plant fibers involves breaking down the hierarchical cellulose structure into small bundles of fibrils [97]. The resulting CNFs’ morphological structures, such as diameter, length, aspect ratio, and surface chemistry, are influenced by the cellulose source, pretreatment, and mechanical or chemical treatments [99].

Mechanical methods are the predominant method for extracting CNFs. These techniques depend on the application of high shear forces to rupture the cellulose fibers and form nanoscale fibrils. Methods include ultrasonication, high-pressure homogenization, microfluidization, grinding, and other mechanical cell-wall refinement techniques [100,101]. This method maintains the chemical structure of cellulose and does not use strong chemicals; however, they consume substantial amounts of energy.

Chemical or enzymatic pretreatments are commonly applied before mechanical fibrillation to enhance efficiency and reduce energy expenditure [102]. For example, alkaline pretreatment can remove hemicellulose and partially delignify the fibers, thereby increasing their swelling and making them more amenable to fibrillation [103]. Oxidative methods, including 2,2,6,6-tetramethylpiperidine-1-oxyl (TEMPO)-mediated oxidation, deposit carboxyl groups on the cellulose surface, thereby increasing the electrostatic repulsion between the fibrils, allowing easy separation under mechanical treatment [104,105]. Combining chemical pretreatment with mechanical fibrillation results in smaller diameters, higher aspect ratios, and more uniform dispersion of CNFs [106].

**Figure 5:**
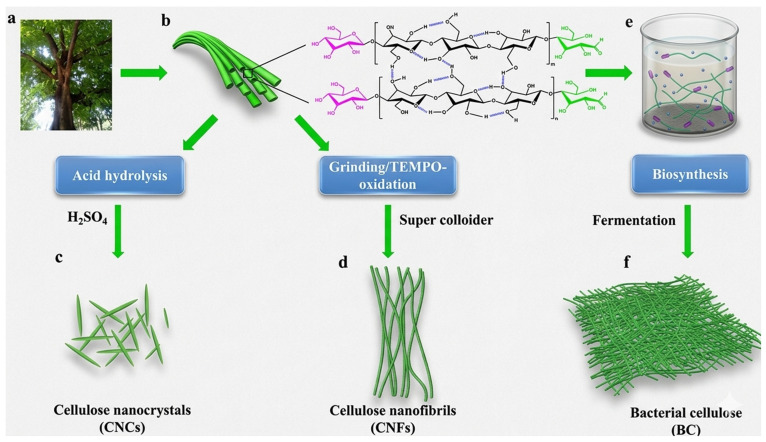
Schematic illustration of the preparation and synthesis process of nanocellulose. (**a**) Plant biomass (**b**) Wood fiber, (**c**) CNC suspension, (**d**) CNF suspension, (**e**) bacteria under static cultivation conditions to produce bacterial cellulose, (**f**) BC pellicle [110].

Enzymatic pretreatments partially hydrolyze amorphous regions of cellulose while reducing the energy required for mechanical defibrillation, softening the fibers, and yielding more uniform CNFs, all without the use of harsh chemicals [107]. Additionally, hybrid methods combining chemical or enzymatic pretreatment with mechanical processing have been reported in CNF extraction studies [108]. Method selection depends on the fiber source, CNF characteristics, and desired application. Optimized extraction methods show cellulose nanofibers with defined morphologies, mechanical strength, high performance, and favorable surface properties, enabling the production of sustainable materials and advanced composites [109]. The schematic diagram in Figure 5 shows the different methods for the production of nanocellulose from plant biomass.

### 3.3. Recycled PET Plastic Powder: Preparation and Production

Recycled PET Plastic Powder is a popular raw material for composite manufacturing owing to its excellent mechanical properties, chemical resistance, and recyclability. PET powder is usually obtained from post-consumer or post-industrial PET materials, such as bottles, packaging films, and textile fibers. The collected materials are first cleaned and sorted to eliminate contaminants, labels, and non-PET parts [111,112]. After this cleaning, PET is shredded into flakes and dried to reduce moisture content, an essential step to prevent hydrolytic degradation during subsequent processing [113]. The flakes can then be melt-extruded and cooled to form solid PET strands or pellets. The strands can then be ground or processed into fine powders for their intended applications [114]. Several methods exist for producing PET powder, with mechanical milling and cryogenic grinding being the most widely adopted methods. The cryogenic process uses liquid nitrogen or other cooling agents to embrittle PET into a finer powder. Grinding PET is difficult at room temperature because PET can either soften or be deformed [115]. Recycled PET powder is thermally stable and retains sufficient crystallinity to be incorporated into polymer composites, powder processes, or additive manufacturing processes.

#### 3.3.1. Mechanical Milling Method for Recycled PET Processing

Mechanical milling is one of the most common methods for reducing PET particle size to fine particles. The PET is first shredded into pellets or flakes before undergoing mechanical grinding, where the particles are further broken down into fine particles via impact and shear forces. The most widely used mechanical grinding method in PET processing is ball milling. Ball milling is widely adopted because the speed, duration, and pause time can be adjusted to reduce excessive heat generation, thermal degradation, or partial melting of PET. However, some problems associated with mechanical milling include variations in the particle size distribution and changes in the polymer microstructure [116].

A study by Lionetto et al. reported that PET pellets or recycled plastic bottles were subjected to dry ultra centrifugal milling, and then passed through sieves of progressively smaller mesh sizes (500, 250, and 80 μm), yielding particle sizes of 100–120 μm. The milled powder particles were then annealed for 4 h at 160 °C to restore brittleness prior to wet ball milling in a zirconia jar with 0.5 mm zirconia balls. Short milling cycles with pauses were followed to control the temperature during milling, which yielded a stable aqueous dispersion of PET nanoparticles with sizes of approximately 70–400 nm [116].

**Figure 6:**
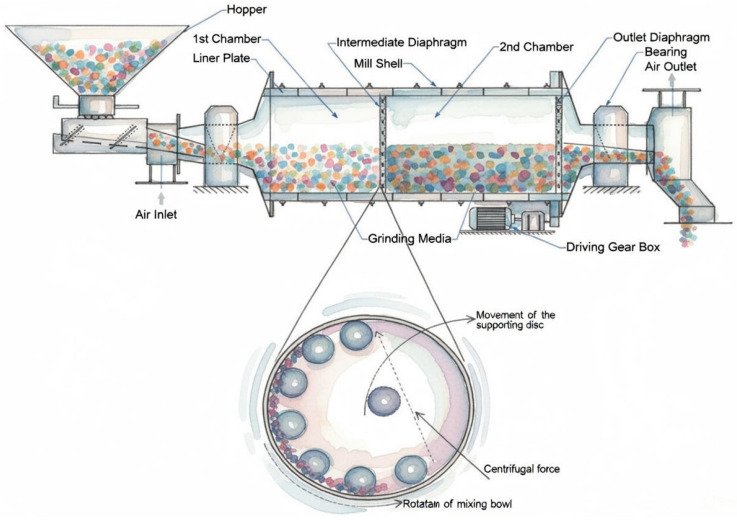
Schematic representation of a typical ball mill grinder. Reproduced from source [118].

Mechanical milling enables the production of PET powders with variable sizes and microstructural characteristics, but control of processing conditions is necessary, as particle-size heterogeneity and changes in crystallinity or microstructure influence the thermal and mechanical behavior of the final PET powders [117]. The following schematic in Figure 6 illustrates the basic design and operation of a typical ball mill grinder commonly used in polymer processing.

#### 3.3.2. Cryogenic Grinding Method

Cryogenic grinding is a high-tech method for producing fine, uniform recycled PET powder while minimizing thermal degradation. In this method, PET flakes are frozen in liquid nitrogen at −150 to −196 °C, which embrittles the polymer, thereby making particle breakage easier during grinding [115,119,120,121]. After embrittlement, PET is ground in a cryogenic mill or an impact grinder with high-speed blades or hammers to reduce the polymer into fine powders. At these low temperatures, grinding produces less frictional heating, which maintains the molecular weight, crystallinity, and thermal stability of polymers [119]. Cryogenic grinding offers several advantages over traditional milling methods. These benefits range from a reduced risk of thermal degradation to improved control in particle morphology and size, enabling a more uniform powder with better flowability, all of which are important for subsequent processing, such as composite production, molding, and powder coating [120,122].

**Figure 7:**
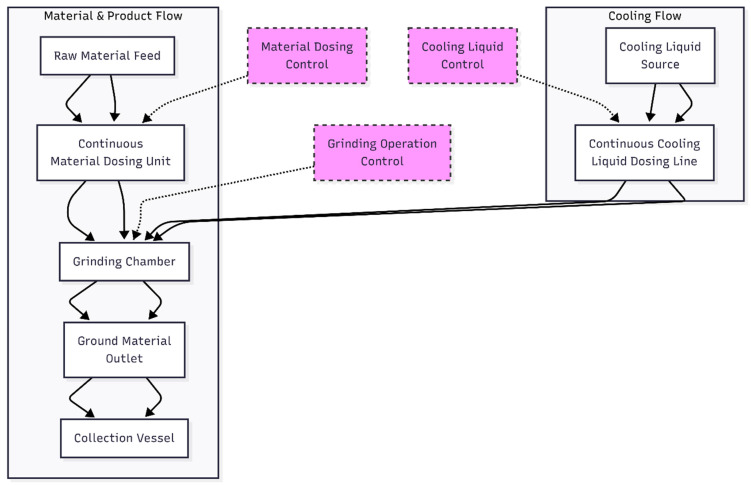
Cryogrinding System for fine grinding of Plastics. Reproduced from source [126].

In one study, PET bottles were shredded and cryomilled in a ball mill cooled with liquid nitrogen at −196 °C. The fine powder obtained by cryogenic milling of PET shows a slight increase in crystallinity compared to the starting material. The results show that the temperature was low enough to preserve the polymer’s thermal properties, such as its melting temperature and stability, due to minimal thermal degradation and limited molecular reorganization during grinding. The powder produced is suitable for applications requiring high thermal performance, such as polymer composites and additive manufacturing [123,124]. Cryogenic grinding typically produces particles of approximately 20–200 µm, and the distribution is generally more uniform and smaller than in normal milling at ambient temperature, enhancing consistency and applicability in composite materials [119,120]. However, cryogenic grinding is not without limitations. The requirement for liquid nitrogen and specialized machines increases the processing costs compared to a typical milling process [125]. However, the increased complexity and high cost are trade-offs for the higher powder quality they produce, especially for high-performance or sensitive applications, such as polymer nanocomposites [125], powder coatings, or applications in which powder rheology, crystallinity, and uniformity may be critical [125]. Figure 7 illustrates the cryogrinding system used for plastic grinding.

### 3.4. Comparative Analysis of the Morphological Properties of Biochar, Nanocellulose, and PET

Morphological properties, including particle size, surface area, porosity, and crystallinity, are key determinants of the performance of biomaterials in soil-stabilization composites. These characteristics are typically evaluated using techniques such as scanning electron microscopy (SEM) for surface topography and X-ray diffraction (XRD) for crystalline structure. This section describes the morphological characteristics of biochar, nanocellulose, and recycled polyethene terephthalate (PET) powder, as reported in peer-reviewed studies, using SEM and X-ray diffraction (XRD).

#### 3.4.1. Biochar SEM and XRD Morphology Properties

**Figure 8:**
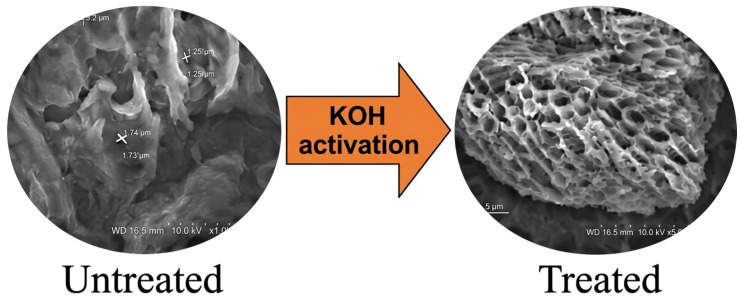
SEM images of untreated and KOH-treated biochar. Reproduced from source [127].

**Figure 9:**
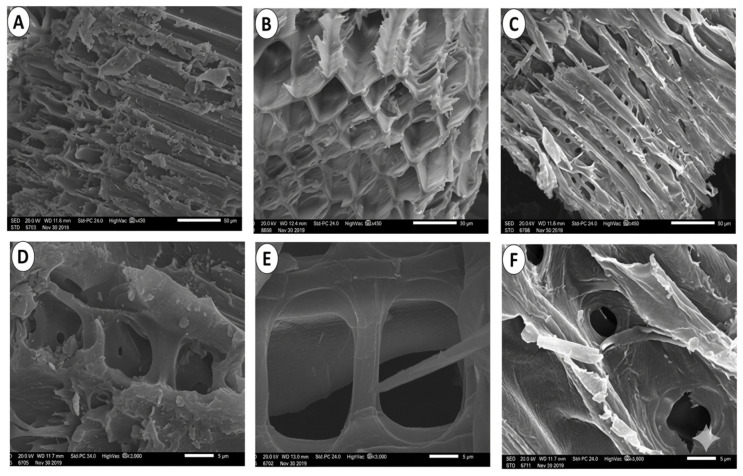
SEM micrographs of samples of Pinewood sawdust biochar (PWSD) (**A**,**D**): biochar activated with steam; (**B**,**E**): biochar; (**C**,**F**): biochar activated with HNO_3_ [128].

**Figure 10:**
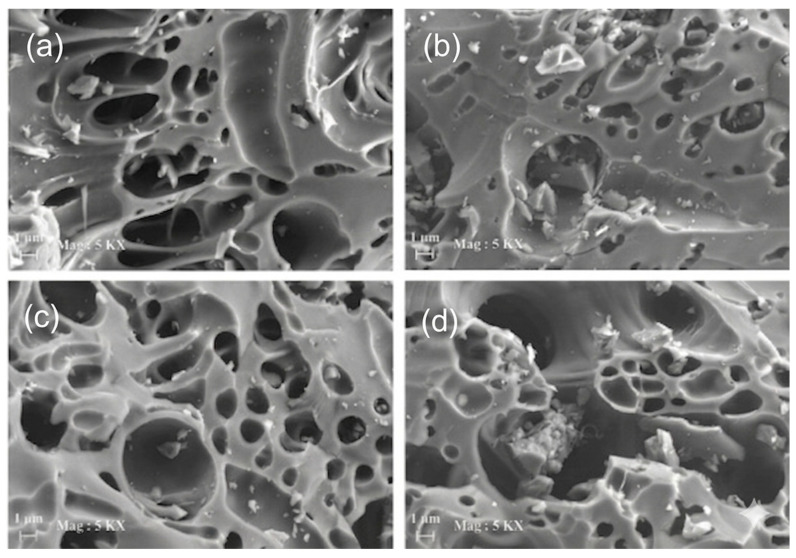
SEM micrographs of (**a**) 0% H_3_PO_4_ activated biochar, (**b**) 45% H_3_PO_4_ activated biochar, (**c**) 65% H_3_PO_4_ activated biochar, and (**d**) 85% H_3_PO_4_ activated biochar [129].

The results of scanning electron microscopy (SEM) analysis of biochar show that activation processes greatly modify its surface morphology and porosity, which have direct application in soil stabilization in road and pavement construction. Untreated biochar tends to have an irregular honeycomb-like structure with macropores and moderate surface roughness inherited from biomass feedstock, providing basic water retention and soil aggregation capabilities; however, chemical activation with KOH enhances micropore and mesopore development to a large extent through intense gas emissions and carbon etching, resulting in a highly fissured rough surface that enhances interfacial bonding with soil particles as seen in Figure 8 [127]. Likewise, steam-activated pinewood sawdust biochar expands the pores into a more open, cavernous network, which promotes drainage while reducing swelling in expansive clays. HNO_3_ activation supplies oxidative pitting and functional groups, increasing nanoscale roughness and chemical affinity for soil minerals, thereby enhancing the resistance to freeze–thaw damage (Figure 9). Progressive H_3_PO_4_ activation demonstrates concentration-dependent pore creation. Low concentrations initiate mesopore formation and surface roughening, optimal levels at 65%, yield a highly interconnected sponge-like structure with maximum surface area for superior capillary water retention and mechanical interlocking in clayey soils. In contrast, high concentrations at 85% may cause pore coalescence or collapse, potentially reducing long-term stability under traffic loading (Figure 10). These structural and morphological benefits, such as increased porosity, surface area, and roughness, enhance soil particle bridging, erosion abatement, crack reduction due to moisture fluctuations, and increased load-bearing capacity. Therefore, activated biochar can be considered an effective, sustainable, and durable soil amendment material [127].

**Figure 11:**
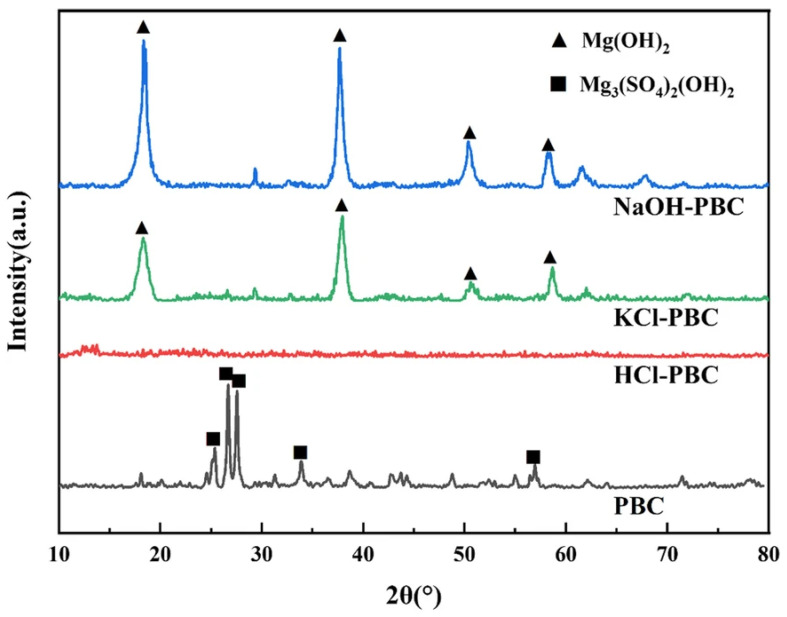
XRD patterns of PBC, HCl-PBC, NaOH-PBC, and KCl-PBC [132].

The XRD patterns illustrate how activation changes the inorganic properties of biochar. Acidic treatments dissolve mineral crystals, expanding the “hump” of the amorphous carbon. Simultaneously, basic or neutral agents can convert or retain specific crystalline phases (e.g., Mg(OH)_2_), which precipitate under these conditions. Figure 11 shows the XRD patterns of a plant-based biochar (PBC) derived from the rice-paper plant (Tetrapanax papyrifer), activated with HCl, NaOH, and KCl, with sample labels (HCl-PBC, NaOH-PBC, and KCl-PBC). Although biochar is non-crystalline [130], the untreated PBC clearly shows distinct diffraction peaks from inorganic salts such as magnesium hydroxide sulfate hydrate (Mg_3_(SO_4_)_2_(OH)_2_) and brucite (Mg(OH)_2_). After activation, HCl-PBC had almost no crystalline phases, indicating that nearly all inorganic impurities were dissolved by acid treatment. In contrast, NaOH-PBC and KCl-PBC exhibited peak activity corresponding to Mg(OH)_2_. This indicates that during alkaline or neutral activation, Mg_3_(SO_4_)_2_(OH)_2_ is transformed into soluble Mg^2+^, which is subsequently reprecipitated as Mg(OH)_2_ [131,132].

#### 3.4.2. Nanocellulose SEM and XRD Morphology Properties

**Figure 12:**
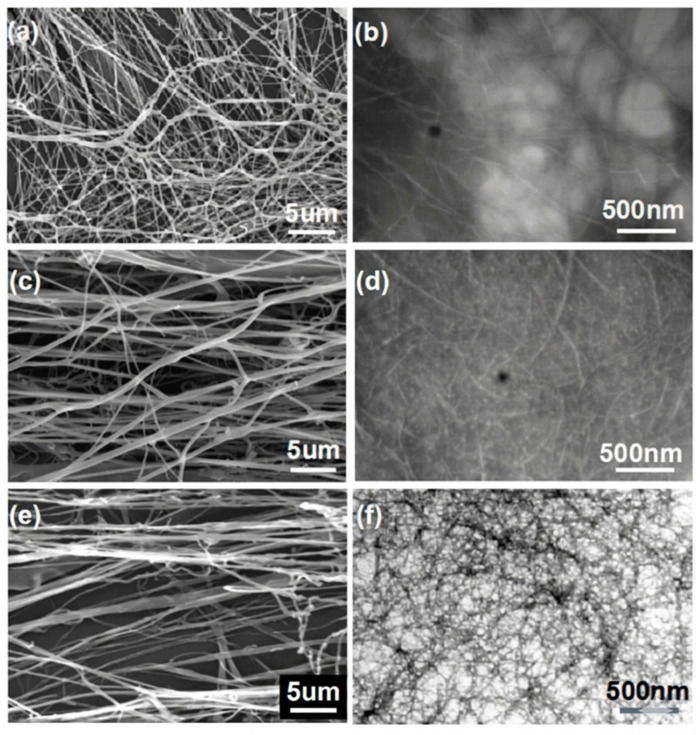
SEM morphology of nanocellulose derived from three different biomass resources: (**a**,**b**) shrub branch; (**c**,**d**) wheat straw; (**e**,**f**) poplar residue [134].

Nanocellulose obtained from lignocellulosic sources exhibits nanofibrillar (CNF) or nanocrystalline (CNC) morphologies based on the biomass used and extraction method. SEM images show elongated, web-like networks or rod-shaped particles that provide enhanced mechanical reinforcement in composites. A study on nanocellulose isolated from palm waste reported that mechanical fibrillation yielded long, amorphous CNF below 100 nm, while acid hydrolysis yielded rod-like CNCs, 7 nm in diameter and 160 nm in length, providing a high aspect ratio for soil binding [133]. Another study compared nanocellulose from three biomass sources processed by grinding and high-pressure homogenization: Amorpha fruticosa (shrub), wheat, and poplar fiber. The shrub yielded the finest, most uniform fibrils with lengths ≥ 5 μm, diameters of 10 nm, and aspect ratios >500. Wheat straw yielded shorter nanofibers with <2 μm in length, 15 nm diameter, and aspect ratios of 100–150, while poplar fiber yielded intermediate structures with lengths < 5 μm, 20–30 nm diameters, and aspect ratios of 150–250 [134]. Figure 12 shows the nanofibrillar structure of nanocellulose obtained from three different biomass.

XRD studies on loblolly pine-derived materials showed that nanocellulose crystallinity index (CrI) plays a significant role in the mechanical reinforcement of nanocomposites. XRD analysis of bleached pulp, cellulose nanocrystals (CNCs), and cellulose nanofibers (CNFs) all retained the cellulose Iβ polymorph and showed Iβ polymorph peaks at 2θ, 15°, 16.5°, 22.5°, and 34.5°. The crystallinity index (CrI) increased from 45% in raw biomass to 74% in bleached pulp due to depletion of amorphous lignin and hemicellulose; and 75–80% for both CNCs and CNFs, but CNFs were marginally higher, having larger crystallite sizes. Factors such as acid concentration, time, temperature, and particle size did not significantly affect the CrI, indicating significant retention of crystallinity across production conditions [135,136].

**Figure 13:**
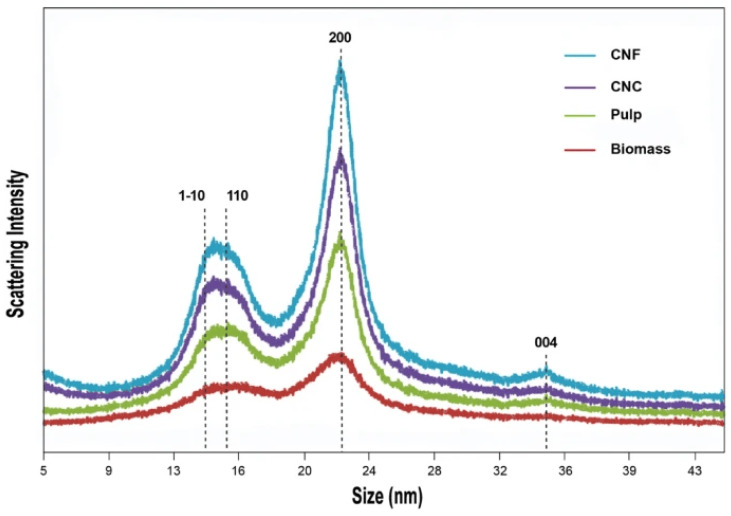
XRD patterns of original biomass (red line), bleached Kraft pulp (green line), CNCs (purple line) from high CNC yielding conditions (i.e., run 4–60% *w*/*w* acid, 60 min, 55 °C, 40 mesh) and CNFs (cyan line) from high CSR yielding conditions (i.e., run 17–54% *w*/*w* acid, 45 °C, 90 min, 20 mesh) [135].

The progressive enhancement in crystallinity from raw biomass to bleached pulp and finally to nanocellulose (CNCs and CNFs) is clearly illustrated in Figure 13, where peak sharpening and increased intensity reflect the effective removal of amorphous components during processing while preserving the cellulose Iβ structure.

#### 3.4.3. Recycled PET Powder’s SEM, XRD, and Morphology Properties

**Figure 14:**
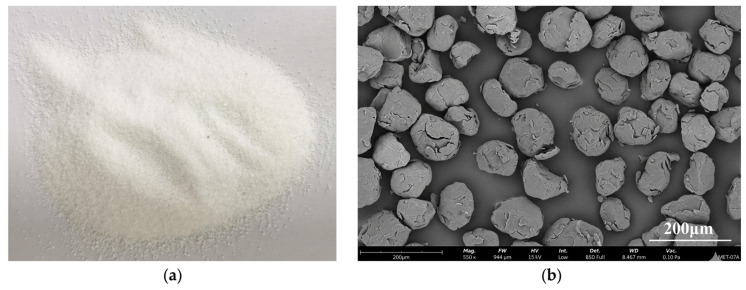
PET powder: (**a**) microscopic morphology and (**b**) SEM image [138].

SEM images of recycled PET powder produced from mechanical or chemical milling show a sharp, irregular, angular particle morphology with rough surfaces and fragmented, elongated shapes. These characteristics increase with longer milling durations, which also promote surface amorphization. The rough texture and irregular geometry enhance interfacial bonding with soil matrices, allowing effective void filling, improved composite cohesion, and greater mechanical interlocking in soil stabilization applications for roads and pavements [116,137]. Figure 14 shows an SEM micrograph depicting the typical irregular morphology of recycled PET powder.

**Figure 15:**
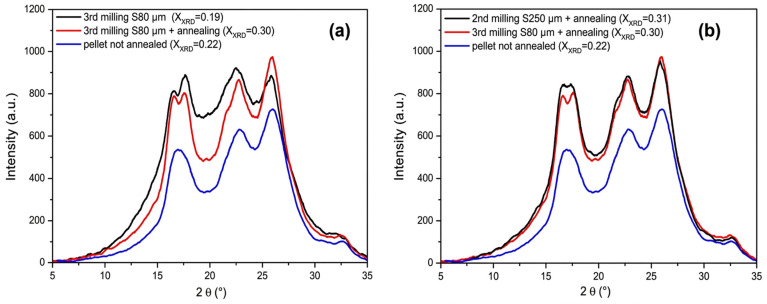
(**a**) Effect of annealing on the XRD patterns of powders milled with the S80 sieve; (**b**) XRD patterns of annealed powders milled with different sieves [117].

X-ray diffraction images also revealed that the morphological and structural properties of recycled PET powder, which are essential for soil stabilization, are strongly influenced by thermal and mechanical treatments. Compared to untreated PET pellets, annealing recovered polymer crystallinity, which would otherwise be diminished during the recycling of non-annealed recycled PET powder processed through an 80 μm sieve [117]. The XRD image is shown in Figure 15.

### 3.5. Mechanical Properties of Biochar, Nanocellulose, and PET

Biochar reinforcement in soil is predominantly via pore refinement, surface roughness enhancement, and physicochemical interactions with soil particles. In contrast, nanocellulose exhibits tensile bridging and elasticity through its hydrogen-bonded fibrillar network. Recycled PET powder is used as a hydrophobic filler and a three-dimensional reinforcement to enhance load transfer efficiency and minimize permanent deformation under repeated loading. This section focuses on unconfined compressive strength (UCS), California Bearing Ratio (CBR), resilient modulus, and swelling-shrinkage mechanical tests from different studies, including expansive clays, clayey soils, and sandy-silty soils. Reported improvements are attributed to better particle interlocking, reduced porosity, pozzolanic properties from biochar, fiber-like reinforcement from nanocellulose, and structural stability offered by porous or fibrous biomaterials, contributing to better bearing capacity under cyclic pavement loading.

#### 3.5.1. Unconfined Compressive Strength (UCS)

UCS is a fundamental indicator of a soil’s ability to withstand compressive loads without lateral confinement, which is critical for assessing subgrade stability. A study reported that biochar produced from the pyrolysis of wood waste at 450–550 °C showed effective performance as a stabilizing agent in expansive clays (EC). The addition of 1–4% fine-grained biochar (FGB) by dry weight increased UCS by up to 31.1% at a 2% mix ratio after 7 days of curing. This improvement was supported by the high porosity and cation exchange capacity of biochar (CEC = > 50 cmol/kg), which promoted water retention, reduced internal friction, and enabled flocculation [11]. Another study explored the use of bamboo biochar (BB) pyrolyzed at 650–700 °C for the stabilization of lean clay (CL)—comprising 73% silt—and silty sand (SM), which contained 52% sand. The biochar was cured for 120 days at 25–27 °C prior to being incorporated. It was observed for the CL soil, UCS increased by up to 10.51% at a 2% bamboo biochar (BB) mix ratio, and for the SM soil, UCS decreased by 21.47% at a 1% BB mix ratio [14].

Another study examined loess-like low-plasticity clay (CL), a composite of wheat straw biochar with pH 7.9, bulk density of 0.5 g/cm^3^, and a polyacrylamide-straw fiber (BPS) mixture. He observed that the UCS increased from 128.63 kPa (control at 28 days) to 565.42 kPa with 3% BPS. The optimal biochar mixing ratio was 2.64. After 28 days, curing was carried out at 20 °C and 95% humidity, resulting in a UCS decline to 429.05 kPa [139].

Nanocellulose, particularly nanocrystalline cellulose (CNC) isolated from wood fibers, serves as a natural stabilizer in sandy-silty soils that are prone to local collapse. Accordingly, 0.5–1.5% CNC addition increased UCS by 30–55%, with the optimum mix observed at 1% [140]. This performance is attributable to the high tensile strength of nanocellulose, up to 7.5 GPa [80], and to hydrogen bonding with soil particles, which form a fibrous network that improves elasticity and reduces local failure.

The use of PET powder, recycled from post-consumer bottles, has also been reported to improve the mechanical reinforcement of clayey soils for pavement bases. A 20–40% increase in UCS was observed with 10–20% PET powder addition. At a water content ratio of 30%, the UCS decreased, indicating a lower compaction density. The low particle size (<0.075 mm) enhances its use as a filler to improve shear resistance in soils [15].

#### 3.5.2. California Bearing Ratio (CBR)

CBR indicates the resistance to soil penetration relative to a standard crushed rock and is used as an empirical index for measuring subgrade strength in pavement designs. In expansive clay (EC) subgrades, the addition of 2% fine-grained biochar (FGB) increased the California bearing ratio (CBR) by 24.1% and reduced the plastic limit (PI), indicating improved aggregate stability [11]. The carbon-rich structure of biochar, typically exceeding 70% elemental carbon, increases the internal surface area and capillary storage capacity, thereby improving moisture absorption under soaked conditions while limiting strength degradation [11,141].

A recent study on coconut shell-derived biochar for expansive soil stabilization reported that the biochar was produced by pyrolysis at 450–550 °C and mixed into the soil at 1–5% by dry soil weight, with an optimum mixing ratio of 2%. They reported that the unsoaked CBR increased from 6.5% to 12.8%, whereas the soaked CBR increased from 3.4% to 7% [141].

In another study, the addition of three biochar types, water hyacinth biochar (WHB), rice husk biochar (RHB), and sugarcane waste biochar (SWB), at 5% by weight into locally available fine-grained soils with clayey and silty particles from Assam, India, was explored. For the silty soil, the CBR increased from an initial value of 1.24% to 3.80% with the WHB, indicating a substantial increase in load-bearing capacity. The changes were small for clayey soil, showing a decline from 3.11% to 3.10% with the SWB. WHB was more effective in silty soils, whereas RHB was more effective in clayey soils [142].

Recent research examined municipal solid waste, such as yam peels and wood biochar, pyrolyzed at 300–450 °C and mixed with lateritic soil at 0–20% by weight. With British Standard Heavy (BSH) compaction, the unsoaked CBR increased from 30.25% to 290.5% at 20% biochar, and the soaked CBR increased from 2.05% to 17.36% at 20% biochar. With British Standard Light (BSL) compaction, the unsoaked CBR increased to 96%, whereas the soaked CBR attained 25% at the optimum mix ratio [143].

Another study reported that CBR values increased by up to 11-fold after the addition of 1.5% nanocellulose to silty sand, and the soil subgrade classification changed from “poor” to “very good” according to the AASHTO criteria. This increase is attributed to the hydrophilic nature of nanocellulose, which improves pore moisture content during compaction to 10–12% [140]. For PET powder-stabilized soils, a different study observed a 15–30% increase in CBR with 10–20% PET powder in clayey soils. This indicates that the soil integrates more effectively with the PET powder, reducing the void ratios and increasing the penetration resistance. The best CBR improvements were observed at 1–2%, beyond which dilution decreases performance [15].

#### 3.5.3. Resilient Modulus (*M_R_*)

The resilient modulus is a measure of soil elasticity under repetitive loading and is fundamental to pavement design. It is defined as follows:

(12) $$M_{R}=k_{1}\cdot {\left(\frac{\sigma_{d}}{\sigma_{3}}\right)}^{k_{2}}\cdot \sigma_{3}^{\;k3}$$

*M_R_* represents the resilient modulus, *σ*_*d*_ the deviator stress, *σ*_3_ the confining stress, and *k*_1_, *k*_2_, *k*_3_ are empirical material constants. Saberian et al. reported a 31.5% increase in resilient modulus for expansive clay (EC) [11]. Another study investigated wood-waste biochar combined with a cationic bitumen emulsion for the stabilization of an AASHTO A-4 laterite road base, characterized as a low-plasticity silt. Biochar was added at a rate of 3–7%, and the emulsion at 3.6–12.6%. The optimal resilient modulus was observed at 7007 MPa dry with 3% biochar and 4.8% emulsion and 5265 MPa soaked with 3% biochar and 4.2% emulsion, greater than Austroads minima of ≥4000 MPa dry, ≥2000 MPa soaked [144]. In another study, the variable resilient modulus was significantly increased by adding 0.5%, 1%, and 1.5% nanocellulose to local soil. $M_{R}$ progressively increased from 138 kg/cm^2^ (control) to 272 kg/cm^2^ at higher mix ratios. This enhancement is due to the development of hydrogen-bonded nanofibrillar networks, which introduce tensile reinforcement, restrict particle mobility, and provide resistance to cyclic deformation [140]. On the other hand, a study on clayey soil stabilization by Carvalho et al. observed 10% PET powder improved $M_{R}$ by 115–247, 20% PET powder improved $M_{R}$ by 79–226, and 30% PET powder improved $M_{R}$ by 60–148 [15].

#### 3.5.4. Swelling and Shrinkage Resistance

The swelling and shrinking of expansive soils due to moisture fluctuations can lead to pavement subgrade failure. The hydrophobic properties of biomaterials enhance moisture management in the soil by refining the pore structure. Fine-grained biochar at a 2% mix ratio improved the swell-shrinkage index by 7%. This is because biochar’s micropores with diameters <2 nm retain water, thereby limiting the expansion of clay minerals [11]. Another study showed that adding sugarcane bagasse biochar at 10% significantly decreased the volumetric shrinkage (VS) in expansive soils from 15.28% in untreated soil to 8.82%. This was the greatest reduction among the tested amendments, outperforming uncharred sugarcane bagasse and both rice husk variants. The improvement is linked to mechanisms that also lower the coefficient of linear extensibility (COLE), demonstrating biochar’s effectiveness in mitigating shrinkage and preventing associated cracking in expansive clay soils [145]. A 15–30% reduction in plasticity index and shrinkage was also observed in nanocellulose-reinforced soil, leading to delayed crack formation during cyclic wet-drying [138]. A minimal increase in swelling was also observed in PET powder-stabilized soils with low contents of approximately 10%, which contributed to improved volumetric stability in clayey soils [15].

The long-term durability of biochar (BC), nanocellulose (NC), and recycled PET composites in soil stabilization is affected by filler structure, mix composition, interfacial bonding, and environmental exposure. Key properties include water absorption, freeze–thaw resistance, thermal stability, and chemical durability.

#### 3.5.5. Absorption of Water and Physical Stability

The incorporation of biochar into expansive clay soils significantly enhances water retention by mitigating soil shrink–swell behavior. Biochar replaces expansive minerals, fills void spaces, and absorbs water molecules, thereby limiting the available water required for clay swelling. Its porous structure and high specific surface area enhance cation exchange and flocculation, reducing soil hydrophilicity, thereby improving soil stability, reducing plasticity, and lowering swelling potential [11].

The water absorption behavior of nanocellulose-based composite materials largely depends on the filler composition and dispersion quality. At the optimum mix ratio, nanocellulose exhibits good dispersion, owing to the formation of a dense hydrogen-bonded matrix, in which water diffusion is restricted, and moisture uptake is minimized. In hydrophilic polymer matrices such as PVA, hydrogen bonding between nanocellulose and the polymer matrix consumes the available hydroxyl groups and creates physical barriers that restrict water infiltration. When the nanocellulose mix is increased beyond the optimum mix ratio, particle agglomeration occurs, leading to defects, porous regions, and coarse microstructures that promote water ingress and reduce the composite performance [146,147]. Several studies have reported an increase in water resistance at optimum nanocellulose mix ratios as well as performance loss at higher mix ratios in reinforced biopolymer systems [146,148]. Agglomeration-associated defects are regarded as serious barriers to the synthesis of high-performance nanocellulose composites.

In contrast, recycled PET enhances moisture resistance, primarily owing to its hydrophobic characteristics. Instead of forming an interconnected network, PET is a water-resistant filler that fills voids within the composite, reducing permeability and limiting moisture transport. However, at low concentrations, it has little impact on the mechanical properties. These mechanisms underlie the differences in reinforcement and water absorption observed between hydrophilic nanocellulose-based systems and hydrophobic PET-based composites [148,149].

#### 3.5.6. Freeze–Thaw Resistance

Research on bamboo biochar-modified soils showed its benefit in improving strength retention during freeze–thaw cycles. The study showed that after 10 freeze–thaw cycles, the soil amended with 7.5% biochar by weight had approximately 15% less Unconfined Compressive Strength (UCS), which is much higher than that of untreated soil, revealing the effectiveness of biochar in stabilizing the soil microstructure [14]. In another study, peanut shell biochar was added to clay soil at different concentrations (0%, 2%, 4%, and 8% by weight), which reduced freeze–thaw effects by changing large pores into smaller micro and mesopores, preserving the soil water retention, especially with higher biochar contents, and keeping hydraulic conductivity stable with different compaction densities and water contents. Thus, these results indicate the ability of biochar to retain hydraulic properties in freeze–thaw-prone clay soils [150,151].

Nanocellulose also improves the freeze–thaw strength of clayey soils. In a recent study, soil samples were obtained from the Yazd-Ardakan desert in Iran and mixed with 0.5–1.5% nanocellulose. The study showed that the reduction in strength loss during repetitive freeze–thaw cycles (up to 10–12 cycles according to ASTM) was significant even at low nanocellulose content. Untreated soils showed rapid loss of strength during the initial cycle due to microstructural damage caused by ice formation, whereas nanocellulose-stabilized samples retained greater residual strength. This improvement is due to the tensile bridging effect in the soil matrix, which maintains particle bonds, limits crack propagation, and reduces water migration during freezing and thawing [138,152,153,154]. Another study reported that 0.05–0.15 wt% cellulose nanofibrils (CNF) can reduce mass loss during freeze–thaw cycling by creating a dense, supportive network that bridges microcracks [152].

Necmi et al. investigated the freeze–thaw resistance of unreinforced and PET fiber-reinforced clayey soil by subjecting samples cured for 7 to 28 days to 10 standardized freeze–thaw cycles, where each 48-h cycle included PET samples frozen at −21 °C and thawed at +21 °C, followed by unconfined compression tests. He reported that the presence of waste PET fibers significantly enhanced the residual strength and durability of soil subjected to cyclic freezing and thawing, compared to unreinforced soil [149]. While no data were found for PET powder, its densification and pore-filling properties could provide positive freeze–thaw benefits [11].

#### 3.5.7. Thermal Stability and Chemical Resistance

Biochar reduces soil heat transfer efficiency and thermal conductivity due to its porous structure, organic composition, and water-holding capacity [155]. A study was conducted to evaluate how biochar application rate, particle size, and soil moisture content affect the thermal properties of sandy soil. The study utilized three mix ratios (0, 1, and 5% *w/w*), three particle sizes (<0.15, 0.15–1, and 1–2 mm), and three water saturation (Sw) levels (0.07, 0.2, and 0.78). It was observed that the application of biochar at a 5% ratio remarkably influenced the physical and thermal behavior of the soil. Smaller biochar particle size (<0.15 mm) was less effective than larger particles in reducing thermal conductivity, resulting in a 21% reduction at low moisture content but 32% for 1–2 mm particles. It was also observed that soil moisture has a greater influence on the thermal properties compared to biochar. Furthermore, the study shows that an increase in moisture content is linearly related to conductivity, diffusivity, and heat capacity, while following a power-law relationship with thermal resistivity [156].

Another study evaluated the thermal conductivity of kaolin clay soil amended with biochar from various feedstocks. The study showed that all soils exhibit a drop in conductivity as they dry. However, a significant difference was observed concerning the feedstock used. Specifically, soil with peach pit biochar exhibited the highest initial conductivity and the steepest decline; reed biochar produced the lowest overall conductivity; and apple wood biochar produced a final conductivity nearly double that of the others at low moisture. The performance differences were attributed to differences in their pore structures. Peach pit and reed biochar both have many distinctive pores that trap insulating air. On the other hand, apple wood biochar has a denser structure with fewer pores. The carbon structure, defined by the feedstock, affects the pore volume and shape. This, in turn, affects the amount of air and water in the soil, thereby altering its thermal properties [157].

Unmodified nanocellulose has significantly lower thermal degradation onset temperatures than cellulose, usually between 200 and 300 °C, due to dehydration, depolymerization, and catalytic action of surface sulfate groups present during isolation. The thermal stability of nanocellulose is affected by factors such as cellulose source, processing method, sulfate content, and crystallinity, which can have profound effects on its thermal properties. Studies show that CNF has better thermal stability than CNC [146,147]. The thermal and moisture resistance can be enhanced by surface modifications, such as acetylation or silylation, which passivate reactive hydroxyl groups [146,147].

Recycled PET shows excellent thermal and dimensional stability owing to its semi-crystalline and hydrophobic nature [149]. Biochar also has high inherent chemical stability due to its aromatic carbon framework, allowing carbon storage for centuries, although surface oxygen groups may enhance reactivity or reduce hydrophobicity [158,159]. However, unmodified nanocellulose is liable to hydrolysis in acidic or alkaline environments. The durability of these materials in soil stabilization presents a clear compromise. Hydrophilic additives such as biochar and nanocellulose enhance initial soil bonding and crack resistance. In contrast, at higher dosages, they may enhance porosity and water retention, potentially compromising long-term stability. In contrast, recycled PET is hydrophobic, restricting moisture penetration and more effectively inhibiting crack propagation under repeated loading cycles [146,154].

#### 3.5.8. Biochar-Nanocellulose-PET Composite Soil Stabilization Optimization

Effective soil stabilization using biomaterial-based composites requires a performance-driven optimization strategy that accounts for soil mineralogy, particle size distribution, and exposure conditions. Rather than relying on universal dosage recommendations, stabilization design must account for how individual biomaterials interact with specific soil fabrics to achieve balanced improvements in strength, durability, and environmental performance. Particle properties, dosage, and soil mineralogy influence soil stabilization performance when incorporating biochar, nanocellulose, and recycled PET powder. Optimum mixing ratios for each biomaterial have been reported in the reviewed studies; beyond these values, mechanical and durability performance deteriorate due to agglomeration, excessive porosity, or weak interfacial bonding. Within a mix ratio of 1–3% by dry soil weight, biochar promotes particle interlocking, moisture regulation, and microstructural stability of the soil [11,141,145]. Nanocellulose exhibits improved performance at <1.5% due to its high surface area, strong hydrogen bonding, and fibrous network formation, which increase strength, stiffness, and shrinkage resistance while retaining elasticity [140,146,152]. Recycled PET powder requires a higher content, typically 10–20%, to function effectively as a filler and reinforcement material, thereby reducing void ratios and improving load transfer. However, excessive mix content introduces discontinuities and reduces the strength and cyclic performance [15,149]. The appropriate composite design will depend on optimizing each biomaterial selection and mix ratio based on soil type, environmental exposure, and desired pavement performance. Fine-grained biochar and PET powder, when used in clayey and expansive soils, inhibited swelling. They provided compressive and resilient properties within the soil [11,141,149], whereas nanocellulose in sandy-silty soils improved cohesion, ductility, and elastic recovery under repeated loading [140,152]. When optimized, a hybrid or ternary system comprising biochar (to refine pores), nanocellulose (to improve tensile strength), and PET (to achieve long-term durability) exhibits promising synergistic effects that enhance its strength, volumetric stability, and environmental resistance [15,146,150].

### 3.6. Potential for Carbon Sequestration in Soil

Biochar, nanocellulose, and recycled PET mitigate climate change through distinct carbon pathways. Biochar acts as a direct carbon sink by physically storing carbon in soils, quantified as long-term CO_2_ sequestration. In contrast, nanocellulose and recycled PET contribute indirectly by displacing carbon-intensive materials such as virgin plastics or cement, yielding avoided CO_2_ emissions.

#### 3.6.1. Biochar Carbon Sequestration in Soil

The effectiveness of biochar as a carbon sink depends on multiple factors, including soil type, biochar feedstock, morphology, mix ratio, and environmental conditions. Numerous studies and meta-analyses have quantified its impact on soil carbon fractions, demonstrating both immediate and long-term benefits for carbon retention and soil performance.

A global meta-analysis conducted by Chagas et al., comprising 169 studies, found that biochar application significantly increased soil carbon fractions, particularly total carbon (64.3%) and organic carbon (84.3%). Maximum sequestration was observed in fine-textured, carbon-poor soils with high biochar application under temperate, controlled conditions. Thus, the biochar effectiveness for climate mitigation is dependent on soil characteristics [160].

A Long-term field study also reported that carbon sequestration by biochar is dependent on soil type. It was reported that biochar application to a loamy soil resulted in a stable and lasting increase in soil organic carbon (SOC) to 38 Mg ha^−1^ over 11 years. In sandy soil, the initial SOC gain of 61 Mg ha^−1^ was nearly completely lost within nine years. The difference could be related to the coarse, porous structure of sandy soils, making added carbon more exposed to microbial decomposition and leaching. The study concluded that long-term storage success depends more on biochar properties and application rates than on other organic additives, and is most reliably achieved in fine-textured soils [161].

Based on a comprehensive global meta-analysis of 75 studies, Bekchanova et al. found that biochar application significantly enhances soil carbon sequestration, with an average increase of 61%. This analysis, which included 250 observations, identified several variables that affect the carbon sequestration property of biochar, including higher biochar application rates, certain feedstock types, and lower pyrolysis temperatures, which were the most effective at promoting carbon storage [162]. Biochar has a condensed aromatic structure that is resistant to microbial degradation, which is the primary reason it stabilizes carbon in soil. Although their persistence depends on the feedstock and pyrolysis conditions, high-carbon biochars act as long-term carbon reservoirs. Their residence time spans centuries, making them far more durable than labile organic matter and more persistent than stable soil organic carbon [160].

Other studies have reported that the carbon sequestration potential of biochar in soil varies between 0.7 and 1.8 Gt CO_2_-C (eq) per year. This was attributed to biochar’s high stability and carbon-to-nitrogen (C/N) ratio, which enabled significant carbon sequestration in the soil. The study also reported that carbon sequestration was enhanced at high biochar application rates in soils with high porosity and alkaline pH (>7.5) [163]. Table 5 shows the biochar’s carbon sequestration potential in soil, as reported in different literature sources.

#### 3.6.2. Nanocellulose Contribution to Carbon Mitigation

The carbon mitigation role of nanocellulose primarily occurs through substituting carbon-intensive additives rather than through direct soil sequestration. Kane et al. conducted life-cycle inventories of 18 nanocellulose production processes to assess greenhouse gas emissions from cellulose nanocrystals (CNCs) and cellulose nanofibers (CNFs). Emissions ranged from 1.8 to 1100 kg CO_2_ eq/kg, depending on the production method. The lowest-emission production pathways identified by the study included mechanical and enzymatic routes, whereas chemical methods produced significantly higher emissions. The study concluded that for nanocellulose to provide net climate mitigation, adoption should prioritize mechanical or enzymatic production methods [164].

A recent study also examined the life cycle assessment (LCA) of CNF and CNC production, demonstrating vast differences in energy consumption and greenhouse gas emissions, influenced by their production methods. He discovered that the chemical pretreatment method has the highest environmental footprint during nanocellulose production. TEMPO-mediated oxidation provided a balanced profile with lower energy demand and global warming potential (GWP). In contrast, carboxylation methods, such as carboxymethylation, require high energy use and are also carbon-intensive. However, there are trade-offs between GWP and water depletion, since methods with lower carbon emissions often require more water. The study concluded that TEMPO oxidation combined with homogenization (TOHO) has the lowest inputs, while methods involving chloroacetic acid etherification and sonification (CESO) have the highest [165]. It is important to note that these values are still significantly lower than those of cement and lime.

While nanocellulose does not yield long-term carbon sequestration in soils, the replacement of fossil-fuel-derived materials with nanocellulose-based alternatives offers a means to minimize dependence on conventional carbon-intensive materials like cement and lime [160]. Comprehensive nanocellulose LCA data comparing production methods and sustainability indicators, such as GWP, fossil fuel depletion, energy use, and other human and aquatic life health indicators, can be found here [165].

#### 3.6.3. Recycled PET Contribution to Carbon Mitigation

A recent study evaluated the environmental performance of PET recycling technologies using a consequential cradle-to-grave life-cycle assessment, comparing the Bottle-to-Fiber and Bottle-to-Bottle pathways. The study reported that PET recycling significantly reduces greenhouse gas emissions by 60% and fossil resource scarcity by 85%. While bottle-to-bottle showed better environmental performance across most impact categories, it yields a lower material circularity indicator (MCI: 0.20–0.31) than bottle-to-fiber recycling (MCI: 0.52) [166]. Life cycle assessments in other studies showed that recycling PET reduces energy use by 84% and GHG emissions by 71% compared to virgin PET production [167].

**Figure 16:**
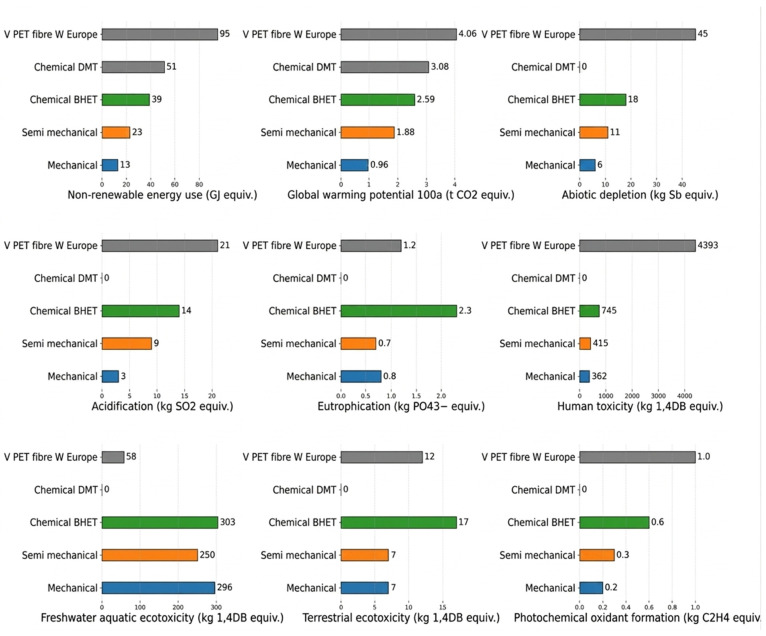
LCA results for 1 ton of recycled PET fiber, based on the “cut-off” approach, for second-life applications, cradle-to-factory gate. Reproduced from source [168].

Another study reported that mechanical recycling of PET provides the best LCA benefits, showing the lowest impact in eight out of nine categories, with the highest savings observed in non-renewable energy use (NREU) and global warming potential (GWP), up to 85% and 76%, respectively. The study also reported that the chemical recycling method (back-to-monomer/oligomer) also reduced NREU and GWD significantly by 24–54%. Categories such as eutrophication and ecotoxicity showed higher impacts due to chemical production [168]. Figure 16 below shows different PET recycling methods and corresponding LCA values.

## 4. Discussion

This section discusses the composite material’s complementary carbon roles and potential environmental concerns, such as soil pollution and microplastic leaching, associated with the use of recycled PET powder, as well as how the presence of biochar and nanocellulose could mitigate these risks. Furthermore, it discusses practical concerns affecting the use of these materials, including optimization, stabilization efficiency, and testing to ensure environmental safety and sustainable application in geotechnical engineering.

### 4.1. Synthesis of Biochar, Nanocellulose, and Recycled PET Carbon Roles

Integrating biochar, nanocellulose, and recycled PET into soil stabilization and composite systems provides complementary pathways for climate mitigation. Biochar provides direct, long-term carbon sequestration in soils, with meta-analyses and field studies demonstrating increases of 60–84% in soil organic carbon that persist for a decade or more, particularly in fine-textured soils [160,161,162,163]. This is due to its condensed aromatic structure, making it resistant to microbial decomposition, thereby providing stable carbon sequestration.

Nanocellulose provides indirect carbon benefits by substituting for carbon-intensive materials such as cement and lime. Low-impact production pathways, such as TEMPO oxidation combined with homogenization (TOHO), provide greater environmental benefits than chemical methods [164,165], thereby promoting net climate mitigation. Recycled PET also generates significant indirect carbon savings by substituting virgin PET production, with Bottle-to-Fiber recycling providing especially large climate benefits. LCA studies show that mechanical recycling has the highest savings in non-renewable energy and global warming potential—up to 85% and 76%, respectively [166,167,168].

The combined use of these materials enables a multi-scale climate mitigation strategy. Each contributes uniquely: biochar provides direct soil carbon sequestration, while nanocellulose and PET introduce carbon savings through avoided emissions by replacing carbon-intensive materials. As a composite, they maximize carbon benefits, reduce demand for emissions-intensive materials, and advance sustainability while improving soil geotechnical performance.

### 4.2. Addressing Soil Pollution and Microplastic Concerns

Soil composites based on biochar, nanocellulose, and recycled PET powder may pose risks to soil and environmental safety, particularly due to microplastics (MPs). Although recycled PET powder is a promising additive, it may contribute to MP accumulation if particles leach into soil during environmental conditions such as weathering, abrasion, or incomplete encapsulation. Soil pollutants can disrupt microbial communities, reduce enzyme activities, impair nutrient cycling, and harm plant growth and ecosystem functions. It is important to understand how these materials might interact in a composite system and how these could help mitigate against these risks.

Biochar’s high surface area, porous structure, and functional groups make it an efficient in situ adsorbent for MPs. Studies indicate that biochar derived from plants can minimize the migration and vertical penetration of MPs in soil porous media, even under wetting-drying cycles simulating seasonal precipitation and evaporation. For instance, 15% biochar can reduce MP escape to approximately 2% in effluent, vs. over 8% without biochar, by providing abundant adsorptive sites, creating tortuous flow paths, and increasing soil aggregation through hydrophilic groups and micropore capillarity, which prevents crack formation and particle flushing [169]. Biochar interacts with MPs via physical adsorption, such as pore filling and surface entrapment within its complex porous network, and chemical adsorption, including electrostatic interactions, hydrogen bonding, and hydrophobic effects. Its high surface area, microporosity, and functional groups, such as carboxyl and hydroxyl groups, provide abundant binding sites, reducing MP mobility in soil porous media. Under cyclic loading, biochar also creates tortuous flow paths and enhances aggregate stability, limiting vertical MP migration and leaching. This immobilization prevents the MPs from dispersing into groundwater or adjacent soils [169,170,171].

Nanocellulose, with its nano fibrils, high aspect ratio, large specific surface area, and abundant hydroxyl groups, absorbs MPs by adsorption through electrostatic attraction, hydrogen bonding, physical filtration, and entrapment in the composite structure, and also through hygroscopic particle trapping in moist soil environments. Within the composite, nanocellulose forms a cohesive, biodegradable binder that envelopes and encapsulates recycled PET powder particles. The hygroscopic nature of nanocellulose allows uniform distribution and binding of biochar particles, which yields a denser, more tortuous matrix that hampers MP transport and migration [172].

### 4.3. Practical Implementation and Standardization Challenges

Although no stabilization system is completely risk-free, as extreme long-term degradation or high additive loading may still present residual risks, evidence from the literature shows that plastic particles can be effectively confined within stabilized soils, leveraging on biochar-mediated immobilization of microplastics and nanocellulose particles capture, as well as synergetic interactions between the component materials, thereby improving performance with limited soil pollution. This confinement can be further improved by optimizing the mix ratio and uniform material mixing to enhance efficient dispersion. Continued long-term field monitoring, including leaching tests and microplastic quantification in geotechnical trials, is still required to fully confirm long-term performance. The interaction mechanisms described in the reviews provide some insight into addressing these concerns.

## 5. Conclusions

This review provides a comprehensive assessment of sustainable soil stabilization using bio-based waste-derived materials, namely biochar, nanocellulose, and recycled PET powder, as potential replacements for cement-based stabilizers. This study presents in-depth performance benchmarks based on extensive mechanical and environmental evaluations across different soil types. The stabilization mechanisms of each additive are clearly evaluated. Biochar plays a significant role in refining pores, regulating moisture, and stabilizing microstructure. It has a highly porous structure, thus facilitating water retention, void filling, and reducing desiccation cracking, aiding in the formation of aggregates that increase volumetric stability and resistance to environmental fluctuations. Nanocellulose improves tensile bridging, interparticle cohesion, and elastic recovery. The nano-fibrils create a hydrogen-bonded network that strengthens the soil fabric, thus improving its tensile strength and recoverable deformation under loading. Recycled PET powder acts as a hydrophobic filler and three-dimensional reinforcement medium, offering mechanical interlocking and void filling, leading to higher load-bearing capacity and long-term durability.

Quantitative analysis of critical geotechnical tests, such as unconfined compressive strength, California bearing ratio, resilient modulus, and volumetric stability, indicates higher performance at optimal mix ratios. Excess mix ratios may cause performance degradation, partly due to particle agglomeration, increased porosity, or weakened interfacial bonding, underscoring the need for performance-driven mix formulation. Soil mineralogy is another important factor influencing performance. Biochar and PET powder generally provide better reinforcement in expansive clay. Meanwhile, nanocellulose yields better reinforcement in sandy and silty soils, especially for controlling plasticity and mitigating shrink–swell behavior. These findings strongly support site-specific, tailored stabilization strategies rather than a one-size-fits-all approach. From an environmental and sustainability point of view, these materials provide huge benefits over conventional stabilizers. Biochar can provide remarkable carbon sequestration, typically ranging from 1.5 to 3 tons of CO_2_-equivalent per ton applied. The use of recycled PET powder diverts post-consumer plastic waste from landfills, thereby minimizing plastic pollution. This leads to reduced greenhouse gas emissions, improved resource efficiency, and enhanced ecosystem preservation, thus positioning them as viable low-carbon alternatives to cement and lime. Their synergistic interactions could also help remediate soil and microplastic pollution. Although biochar and nanocellulose are effective in reducing microplastic risk in soil—because biochar adsorbs microplastics via its porous structure and functional groups, while nanocellulose acts as a binder that encapsulates and traps microplastics such as PET particles, resulting in a dense composite material that limits microplastic migration in soil—there is still a need for further field tests and evaluation prior to adoption.

Regarding field readiness, biochar could be applied at 3–5% by weight and nanocellulose at 0.5–1.5% for pilot-scale implementation in low-traffic, low-risk settings such as rural roads, embankments, and slope stabilization projects. These dosages could offer moderate strength gains, effective plasticity control, and improved shrink–swell resistance in cohesive soils. Recycled PET-based systems, however, warrant greater caution. Their long-term durability, potential environmental concerns, and overall sustainability require further validation before broad adoption. The complete composite system including recycled PET is most appropriately reserved for contained, non-invasive engineering applications where migration risks can be minimized such as: pavement and road subgrades, railway sub-ballast and subgrade layers, building foundations and floor slabs, engineered embankments and structural fills, retaining wall backfills, landfill liner support and cover systems, low-permeability barriers and cutoff walls, industrial yards and logistics platforms, and low-volume rural roads and temporary construction working platforms.

Despite preliminary evidence showing potential synergy in these composites, including enhanced encapsulation, adsorption, and combined reinforcement, there is still limited empirical data regarding multi-additive composite systems. To maximize the value of these low-carbon materials at scale, several steps are essential. Extensive field validation trials with long-term performance monitoring will be required in addition to the development of standardized testing protocols and comprehensive environmental monitoring programs. Controlled factorial experiments, coupled with advanced microstructural characterization, should be prioritized to better understand interaction mechanisms and optimize composite formulations. Lastly, rigorous durability testing under real environmental and mechanical loading conditions is necessary to ensure long-term reliability.

Although this review used a systematic and transparent methodology, several limitations are worth noting. This includes considerable heterogeneity in experimental designs, material properties, and reporting details across studies, thereby severely limiting direct comparisons and transferability. Wide differences between soil classification, mineralogy, moisture content, compaction energy, curing duration, and environmental conditions make cross-study comparisons difficult. Also, the lack of standardized testing protocols and reporting frameworks, as well as inconsistent curing regimes, testing parameters, and evaluation methods, hinders the development of integrated design guidelines and prevents rigorous quantitative meta-analysis. These limitations highlight the need for standardized experimental procedures, expanded field trials, improved reporting practices, and integrated environmental assessment frameworks. Addressing these gaps is essential to enhance data reliability and support the practical adoption of sustainable, waste-based soil stabilization technologies.

## Figures and Tables

**Table 1:** Sustainable supplementary materials and their sources.

Material	Source/Feedstock	Stabilization Mechanisms	Key Benefits	Treated Soils	References/Links
Biochar	Agricultural, municipal, and forestry waste	Fills pores, improves friction, adsorbs moisture, and carbon fixation	Higher shear strength, reduced swelling, improved compaction	Expansive clays and silts	[18]
Lignin/Lignosulfonates	Paper and pulp	Natural binder via cation exchange, polymer film	Increases UCS, reduces plasticity, enhances durability	Clayey, subgrade soils	[19]
Nanocellulose (CNF, CNC, MFC)	Wood pulp	Fiber bridging and bonding	Improved ductility, cohesion, and tensile strength	Silt, sand, fine soils	[20]
Cellulose Fibers/MCC	Paper, plant fibers	Mechanical fiber reinforcement	Better ductility and shrinkage resistance	Silty soils, loose sands	[21]
Natural Fibers (Jute, Coir, Hemp)	Plant fibers	Mechanical reinforcement	Enhanced shear strength, ductility, and reduced settlement	Weak subgrade, coastal clays	[22]
Rice Husk Ash	Rice milling residue	Pozzolanic reaction	Reduced plasticity, higher CBR, improved strength	Clayey soils, soft subgrades	[23]
Sugarcane Bagasse Ash	Sugar industry waste	Pozzolanic reaction, filler	Higher CBR, reduced shrink–swell, increased stiffness	Expansive clays and soft subgrades	[24]
Palm Oil Fuel Ash	Palm oil waste	Pozzolanic and pore refinement	Improved compressive strength and workability	Tropical and lateritic soils	[25]
Corncob Ash	Agri residue	Pozzolanic, granular improvement	Higher bearing capacity, better compaction	Clayey soils	[26]
Recycled PET	Post-consumer plastic bottles	Mechanical interlocking and filling	Improved UCS, CBR, and lower permeability	Expansive clays, silty and sandy soils,	[27]
Banana/Coconut Fiber	Plant fibers	Fiber reinforcement	Better ductility, reduced cracking, improved fatigue	Pavement subgrades	[28]
Wheat Straw Ash	Crop residue	Pozzolanic, filler	Higher strength, durability, and reduced swelling	Clay-rich soils	[29]
Bamboo Biochar/Fiber	Bamboo residues	pore distribution and mechanical reinforcement	Higher strength, lower compressibility, improved stiffness	Silt, clay, fine soils	[30]

**Table 2:** Physically activated biochar properties and CO_2_ adsorption capacity.

Biochar/Carbon Feedstock	Activation/Treatment Method and Conditions	Reported CO_2_ Adsorption Capacity (≈25–298 K, 1 bar)	References
Vine shoots	CO_2_ activation at 800 °C, 0.1 MPa, burn-off 22–26%	1.02 mmol/g (44.9 mg/g) at 25 °C, 14 kPa	[45]
Wheat straw pellets	CO_2_ activation at 800 °C, 0.1 MPa, burn-off 20–29%	1.00 mmol/g (44 mg/g) at 25 °C, 14 kPa	[45]
Wood pellets	Steam activation at 850 °C, 1 h under N_2_ with steam flow of (10 mL/h (water)	0.8–1.0 mmol/g (38.3 mg/g) at 25 °C, 1 bar	[46]
Bamboo charcoal	KOH activation (1:1 ratio) at 700 °C, 1 h under N_2_	3.3–3.5 mmol/g (148.7 mg/g) at 25 °C, 1 bar	[47]
Populus nigra wood	Steam-assisted slow pyrolysis at 600 °C, N_2_/steam mixture	1.12 mmol/g (49.3 mg/g) at 25 °C, 1 bar	[48]
Cellulose fibers	Steam-assisted slow pyrolysis at 700 °C, N_2_/steam mixture	2.33 mmol/g (102.5 mg/g) at 25 °C, 1 bar	[48]
Olive Stone	Steam activation at 800 °C, one h, varying particle sizes (1.4–2 mm optimal)	0.7–1.0 mmol/g (32.1 mg/g)	[49]
Almond shells	Single-step air oxidation at 500 °C with 3–5% O_2_	2.10 mmol/g (92.4 mg/g) at 25 °C, 1 atm	[50]
Pistachio shells	Slow carbonization (pyrolysis) at 720 °C under ambient conditions	3.29 mmol/g (144.8 mg/g) at 25 °C, 5.682 bar	[51]
Nut shells	MDEA amination functionalization at 30 °C, 8 h (after physical/chemical activation with H_2_O vapor and H_3_PO_4_ at 400–500 °C)	3.9–4.0 mmol/g (175.1 mg/g) at 30 °C, 1 bar	[52]

**Table 3:** Chemically activated biochar properties and CO_2_ adsorption capacity.

Biochar Type/Source Material	Activation Agent	Key Properties	CO_2_ Adsorption Capacity	Conditions	Source
Woodchip	KOH	Very high surface area and optimized microporosity via RSM-CCD	9.89 mmol/g (Langmuir Qmax)	0 °C, 1 bar	[62]
Waste distiller’s grains	KOH	Ultra-microporous, SBET ≥ 2800 m^2^/g, pore size 0.6–0.8 nm	8.21 mmol/g	0 °C, 1 bar	[63]
Coconut shell	KOH	Benchmark high-surface-area activated carbon	6.04 mmol/g	0 °C, 1 bar	[64]
Sawdust	KOH + NH_3_	N-doped, strong chemisorption + ultra-microporosity	7–8 mmol/g	25 °C, 1 bar	[65]
Vine shoots	KOH	Highly microporous, excellent regenerability	5.4–6.1 mmol/g	0 °C, 1 bar	[66]
Corn cob	KOH	SBET 2500 m^2^/g, outstanding cyclic stability	6.36 mmol/g	0 °C, 1 bar	[67]
Palm kernel shell	KOH	Low ash, high micropore volume	2–3 mmol/g	25 °C, 1 bar	[68]
Rice husk	KOH + ZnCl_2_	Hierarchical micro/mesoporous structure	4–6 mmol/g	0 °C, 1 bar	[69]
Bamboo	KOH (various ratios)	BET up to 2000 m^2^/g, dominant role of narrow micropores	7.0 mmol/g	0 °C, 1 bar	[70]
Sewage sludge	KOH	High inherent mineral content, fluidizable after SiO_2_ coating	4 mmol/g	25 °C, 1 bar	[71]
Sugarcane bagasse	ZnCl_2_	Well-developed mesoporosity and rapid kinetics	1–2 mmol/g	25 °C, 1 bar	[72]
Coffee grounds	KOH	Microporous carbon; KOH samples show superior textural development.	3 mmol/g	25 °C, 1 bar	[73]

**Table 4:** Chemical composition of some common cellulosic substances [98].

Material	Cellulose	Hemicellulose	Lignin	Ash	Extractives
Algae (green)	20–40	20–50	–	–	–
Cotton, flax, etc.	80–95	5–20	–	–	–
Grasses	25–40	25–50	10–30	–	–
Hardwoods	45 ± 2	30 ± 5	20 ± 4	0.6 ± 0.2	5 ± 3
Hardwood barks	22–40	20–38	30–55	0.8 ± 0.2	6 ± 2
Softwoods	42 ± 2	27 ± 2	28 ± 3	0.5 ± 0.1	3 ± 2
Softwood barks	18–38	15–33	30–60	0.8 ± 0.2	–
Cornstalk	39–47	26–31	3–5	12–16	–
Wheat straw	37–41	27–32	13–15	11–14	–
Newspaper	40–55	25–40	18–30	–	–
Chemical pulp	60–80	20–30	2–10	–	–
Sorghum stalks	27	25	11	–	–
Corn stover	38–40	28	7–21	3.6–7.0	–
Coir	36–43	0.15–0.25	41–45	2.7–10.2	–
Bagasse	32–48	19–24	23–32	1.5–5	–
Rice straw	28–36	23–28	12–14	14–20	–
Wheat straw	33–38	26–32	17–19	6–8	–
Barley straw	31–45	27–38	14–19	2–7	–
Sorghum straw	32	24	13	12	–
Sweet sorghum bagasse	34–45	18–28	14–22	–	–

**Table 5:** Impact of biochar and soil properties on soil organic carbon sequestration potential [163].

Biochar Synthesis Conditions	Types of Studies	Soil Type	Biochar Application Rate	Carbon Sequestration Potential
Corn silage, 500 °C	Lab-scale	Clay-sandy	1% (*w*/*w*)	no impact on forest soil, but reduced CO_2_ emission from the soil
Swine manure, 600–800 °C	Lab-scale	Silt	2% (*w*/*w*)	Significant reduction of CO_2_ emissions after biochar treatment
Reed straw, 400 °C (nZVI-biochar)	Lab-scale	Saline-alkali soil	0.15–0.45% (*w*/*w*)	Significant reduction of CO_2_ emissions after nZVI-biochar treatment
Corn cob, 250 °C	Lab-scale	Acidic sandy soil	∼0.84% (*w*/*w*)	Reduction of CO_2_ emissions by 11.8%
Hard wood, 200–600 °C (Steam and CO_2_ activation)	Lab-scale	Topsoil (silty soil)	0.75% (*w*/*w*)	Reduction of CO_2_ emissions by 18%
Wood sawdust, 450 °C	Lab-scale	Silty sand	3.2% (*w*/*w*)	Negative priming effects were observed with biochar treatment (−0.22 to −23.56 mg-CO_2_–C/g -soil-C)
Rice straw, 500 °C	Lab-scale	Saline–Alkaline Soil (sandy soil)	~0.77% (*w*/*w*)	Reduction of CO_2_ emissions by 35.19% with the addition of biochar, as well as straw and urea
Wheat straw, 450 °C	Lab-scale	Irrigated Anthrosols (Silty clay)	∼1.1% (*w*/*w*)	Biochar application decreased CO_2_ emissions by an average of 23%
Rice husk, 300 °C	Lab-scale	Soil of Bungor Series (Silty clay)	∼0.54% (*w*/*w*)	Cumulative CO_2_ emissions reduced by 139.41% compared to the control
Wheat straw, 500–600 °C	Pot experiments	Clay soil	50–95% (*w*/*w*)	CO_2_ emissions reduced by 8.05–31.46%. Higher CO_2_ emissions were observed at higher biochar doses.
Pine wood, 500–700 °C	Pot experiments	Clay soil	1% (*w*/*w*)	Reduced CO_2_ emissions by 66.9–72.4%
Corn stalks, 400 °C	Microcosms	Coastal saline soil	16 tons/hectare	Corn stalk-derived biochar showed higher GWMP (−3.84 to −3.17 tonne CO_2_-eq/hectare/tonne C) than the control treatment (−0.11 tonne CO_2_-eq/hectare/tonne C).
Multiple feedstocks, 280 °C	Pot experiments	Alkaline clay and acidic sandy soil	4 tons/hectare	Biochar with N fertilizer addition reduced CO_2_ emission by 7–12%
Corn straw, 360 °C	Microcosm	Sandy clay	9 tons/hectare	Reduced CO_2_ emissions by 11%
Farm waste and wood residues, 500–550 °C	Field-scale	Sandy	11 tons/hectare	Biochar amendment reduced CO_2_ fluxes. However, no significant differences in CO_2_ emission rates among different types of biochar treatments
Corn cobs, 500–550 °C	Field-scale	Sandy soil	0–30 tons/hectare	Specific maintenance respiration (qCO_2_) reduced by 66–73%
Maize straw, 350–550 °C	Field-scale	sandy soil	30 tons/hectare	Reduced CO_2_ emissions by 33%
Corn straw, 450 °C	Field-scale	Sandy soil	20 tons/hectare	Biochar addition enhanced SOC levels and reduced CO_2_ emissions

## Data Availability

No new data were created or analyzed in this study. Data sharing is not applicable to this article.

## Abbreviations

The following abbreviations are used in this manuscript:

AASHTO	American Association of State Highway and Transportation Officials
BET	Brunauer–Emmett–Teller
BB	Bamboo Biochar
BHET	Bis-Hydroxy Ethylene Terephthalate
BNC	Bacterial Nanocellulose
BPS	Biochar-Polyacrylamide-Straw Fiber
BSH	British Standard Heavy
BSL	British Standard Light
CBR	California Bearing Ratio
CEC	Cation Exchange Capacity
CED	Cumulative Energy Demand
CEHO	Chloroacetic Acid Etherification + Homogenization
CESO	Chloroacetic Acid Etherification + Sonication
CL	Lean Clay
CNF	Cellulose Nanofibers
CNC	Cellulose Nanocrystals
CO_2_	Carbon Dioxide
COLE	Coefficient of Linear Extensibility
CrI	Crystallinity Index
DMT	Dimethyl Terephthalate
EC	Expansive Clays
FGB	Fine-Grained Biochar
GHG	Greenhouse Gas
GGBS	Ground Granulated Blast-Furnace Slag
Gt	Gigaton
GWP	Global Warming Potential
LCA	Life Cycle Assessment
MCC	Microcrystalline Cellulose
MCI	Material Circularity Indicator
MFC	Microfibrillated Cellulose
MP	Microplastics
NREU	Non-Renewable Energy Use
PET	Polyethylene Terephthalate
POY	Partially Oriented Yarn
PRISMA	Preferred Reporting Items for Systematic Reviews and Meta-Analysis
PVA	Polyvinyl Alcohol
RHA	Rice Husk Ash
RHB	Rice Husk Biochar
SEM	Scanning Electron Microscopy
SM	Silty Sand
SOC	Soil Organic Carbon
SWB	Sugarcane Waste Biochar
TEMPO	2,2,6,6-Tetramethylpiperidine-1-Oxyl
TOHO	TEMPO Oxidation + Homogenization
TOSO	TEMPO Oxidation + Sonication + Centrifuge Purifying
UCS	Unconfined Compressive Strength
VS	Volumetric Shrinkage
WHB	Water Hyacinth Biochar
XRD	X-Ray Diffraction
